# Intact Memory for Irrelevant Information Impairs Perception in Amnesia

**DOI:** 10.1016/j.neuron.2012.05.014

**Published:** 2012-07-12

**Authors:** Morgan D. Barense, Iris I.A. Groen, Andy C.H. Lee, Lok-Kin Yeung, Sinead M. Brady, Mariella Gregori, Narinder Kapur, Timothy J. Bussey, Lisa M. Saksida, Richard N.A. Henson

**Affiliations:** 1 Department of Psychology University of Toronto, Toronto, ON M5S 3G3, Canada; 2 Rotman Research Institute Toronto, ON M6A 2E1, Canada; 3 Department of Psychology University of Amsterdam, Amsterdam 1018 XA, The Netherlands; 4 Department of Experimental Psychology University of Oxford, Oxford OX1 3UD, UK; 5 Department of Experimental Psychology, University of Cambridge, Cambridge CB2 3EB, UK; 6 MRC and Wellcome Trust Behavioural and Clinical Neuroscience Institute, University of Cambridge, Cambridge CB2 3EB, UK; 7 Research Department of Clinical, Educational and Health Psychology, University College London, London, WC1E 7HJ, UK; 8 MRC Cognition and Brain Sciences Unit, Cambridge CB2 7EF, UK

## Abstract

Memory and perception have long been considered separate cognitive processes, and amnesia resulting from medial temporal lobe (MTL) damage is thought to reflect damage to a dedicated memory system. Recent work has questioned these views, suggesting that amnesia can result from impoverished perceptual representations in the MTL, causing an increased susceptibility to interference. Using a perceptual matching task for which fMRI implicated a specific MTL structure, the perirhinal cortex, we show that amnesics with MTL damage including the perirhinal cortex, but not those with damage limited to the hippocampus, were vulnerable to object-based perceptual interference. Importantly, when we controlled such interference, their performance recovered to normal levels. These findings challenge prevailing conceptions of amnesia, suggesting that effects of damage to specific MTL regions are better understood not in terms of damage to a dedicated declarative memory system, but in terms of impoverished representations of the stimuli those regions maintain.

## Highlights

► Perception can be impaired in amnesia resulting from perirhinal cortex (PRC) damage ► Reducing object perceptual interference can rescue PRC-damaged amnesics' performance ► Memory loss after PRC damage can reflect heightened susceptibility to interference ► Impoverished object representations may contribute to amnesia

## Teaser

Barense and colleagues demonstrate that perceptual deficits in amnesic patients with medial temporal lobe damage can be rescued by reducing interference from visually similar stimuli. These findings challenge current views on amnesia and the neural basis of memory.

## Introduction

Memory loss following brain damage, for example to structures in the medial temporal lobe (MTL), is often considered to reflect a failure to consolidate memory traces that otherwise decay. Recently, however, there has been a resurgence of interest in the idea that amnesia results from increased susceptibility to interference from intact, but irrelevant, memories (Bartko et al., 2010; Cowan et al., 2004; Della Sala et al., 2005; Dewar et al., 2009; Loewenstein et al., 2004; McTighe et al., 2010; Wixted, 2004). Notably, this idea was proposed over 40 years ago (Warrington and Weiskrantz, 1970) but was later largely rejected (Warrington and Weiskrantz, 1978). Moreover, the deficits that follow MTL damage are classically believed to be restricted to memory, and many still argue this to be the case (Clark et al., 2011; Kim et al., 2011; Squire and Wixted, 2011; Squire and Zola-Morgan, 1991; Suzuki, 2009), in spite of recent reports suggesting that perception may also be compromised (Barense et al., 2007, 2010b, 2011b; Bartko et al., 2007; Baxter, 2009; Buckley et al., 2001; Lee et al., 2005a, 2005b; Lee and Rudebeck, 2010). A recent representational-hierarchical account unites these findings, suggesting that apparently distinct mnemonic and perceptual functions may arise from common representations and computational mechanisms.

**Figure 1:**
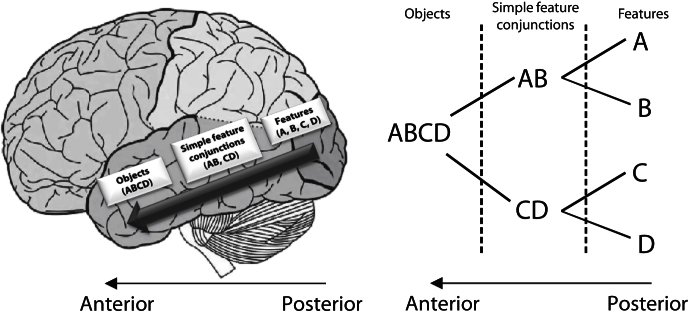
Representational-Hierarchical Theory (A) Lateral view of the human cerebral cortex demonstrating the ventral visual stream (VVS) object processing pathway according to the representational-hierarchy theory (Cowell et al., 2010a). The perirhinal cortex is proposed to reside at the apex of this processing pathway, containing complex representations of objects. (B) The proposed organization of visual object representations in the VVS. A, B, C, and D refer to relatively simple object features represented in posterior regions. More complex conjunctions of these features are stored in more anterior regions, including perirhinal cortex. Figure adapted from Bussey and Saksida (Bussey and Saksida, 2002).

The representational-hierarchical account proposes that the perirhinal cortex (PRC) can be considered an extension of the representational hierarchy within the ventral visual stream (VVS) (Barense et al., 2005; Bussey and Saksida, 2002; Bussey et al., 2002; Desimone and Ungerleider, 1989; Graham et al., 2010; Riesenhuber and Poggio, 1999). It is well-established that as information flows from posterior to anterior regions of the VVS, representations of visual stimulus features are organized hierarchically in increasingly complex conjunctions (Figure 1; Desimone and Ungerleider, 1989; Riesenhuber and Poggio, 1999; Tanaka, 1996). When an object is viewed, multiple representations of this object are activated throughout the entire VVS, with different representations occurring at different stages of the pathway. The object's low-level features are represented in early posterior regions, whereas conjunctions of features are represented in more anterior regions, with the most complex feature conjunctions—perhaps at the level of the whole object—being represented in regions such as the PRC. The traditional memory systems view argues that MTL structures such as PRC support exclusively mnemonic functions (Clark et al., 2011; Kim et al., 2011; Squire and Wixted, 2011; Squire and Zola-Morgan, 1991; Suzuki, 2009). In contrast, the representational-hierarchical view proposes that stimulus representations throughout the VVS and MTL are useful for any cognitive function that requires them (Bussey and Saksida, 2002; Cowell et al., 2006, 2010a). This account seeks to explore whether damage to the high-level representations maintained in MTL regions can account for a variety of deficits observed in amnesia. Under this model, one need not postulate separate memory and perceptual systems. One important prediction of this view—yet to be tested in humans—is that if the complex, object-level representations within the PRC are damaged, interference from incidental, irrelevant features can become catastrophic (Cowell et al., 2006; McTighe et al., 2010). A stream of visual input (such as that encountered over a delay) can create interference at the level of individual features, simply because different objects tend to share lower-level features (e.g., shapes, colors, etc.). However, the *conjunctive* representations usually maintained in PRC are unique to each individual object and resolve this interference. A similar argument would apply to other regions in the MTL such as the hippocampus, albeit in the context of more complex stimulus representations such as spatial scenes (Bussey and Saksida, 2007; Cowell et al., 2010a; Lee et al., 2005a, 2005b).

**Figure 2:**
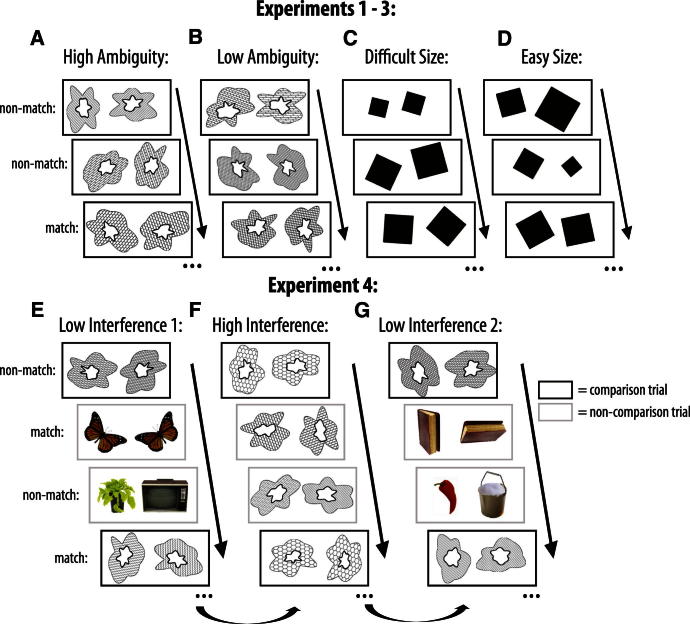
Visual Discrimination Task Participants indicated whether two simultaneously presented stimuli were a match or a non-match. For experiments 1–3, there were four conditions: (A) High Ambiguity Objects, (B) Low Ambiguity Objects, (C) Difficult Size, (D) Easy Size. The objects were defined by three features: inner shape, outer shape, and fill pattern. For High Ambiguity nonmatch trials, only one of these three features differed, whereas for Low Ambiguity nonmatch trials, all three features differed. Thus, the High Ambiguity Object condition placed a greater demand on high-level conjunctive representations and analysis of the object as a whole, which was confirmed by an analysis of eye movement patterns (see Experiment 1). In the Size control task, participants decided if two rotated squares were the same size. (E–G) Example stimuli and trial order from the Low and High Interference conditions in experiment 4. For the Low Interference condition, a High Ambiguity Object trial was always followed by two trials involving perceptually distinct, colored objects (30 High Ambiguity Object trials in total). The High Interference condition was a straight block of 88 consecutive High Ambiguity Object trials. To avoid confounding effects of fatigue, the order of testing conditions was: Low Interference 1, High Interference, Low Interference 2. We compared performance on every third trial only (black boxes), thus ensuring that for each condition our comparison trials were 30 High Ambiguity Object trials with matched stimulus schedules. Across all experiments, all objects were trial unique, though the individual features (e.g., shape segments, fill patterns) repeated across trials.

To test this idea for the first time in humans, we focused on PRC as a structure located at the interface between putative mnemonic and perceptual systems in the brain. Thus, we concentrated on the type of visual objects thought to be represented in PRC (e.g., Barense et al., 2005; Bussey et al., 2002) and developed a visual matching task in which participants indicated whether two simultaneously presented trial-unique objects were the same or different (Figures 2A–2D). Across the different conditions, we manipulated the degree to which conjunctions of object features would be processed. In the High Feature Ambiguity condition, many features overlapped across objects and thus the overall object conjunction (as opposed to single features) provided a more efficient analysis strategy. In the Low Feature Ambiguity condition, a single feature readily provided the solution. Two size conditions provided a control for task difficulty. Experiment 1 investigated eye movements in healthy participants to determine participants' underlying strategy for solving the discriminations (i.e., using single features versus conjunctions). In experiment 2, we used fMRI of healthy participants to test the following two predictions: (1) activity within the PRC would be modulated by the degree of feature ambiguity, when controlling for difficulty, and (2) this modulation by feature ambiguity would be greater in the PRC than in a neighboring MTL area, the hippocampus. While the hippocampus is also implicated in amnesia, its function according to the representational-hierarchical theory is to bind objects to spatiotemporal contexts, not to bind features into objects (Cowell et al., 2006, 2010a; see also Diana et al., 2007; Lee et al., 2005a, 2005b), and thus we would not expect hippocampal activity to be modulated by degree of feature ambiguity using objects.

In experiment 3, we administered the same task to six amnesic cases with focal brain damage and similar degrees of memory impairment. Based on structural and volumetric analyses of critical regions within the MTL, these cases were categorized as follows: (1) individuals with bilateral medial temporal lobe damage that included PRC (MTL cases with PRC damage: n = 2) and (2) individuals with damage predominantly limited to the hippocampus (HC cases: n = 4). We predicted (1) worse performance in the MTL cases with PRC damage relative to healthy controls, specifically for the High Ambiguity condition, and (2) a greater such impairment in the MTL cases with PRC damage than HC cases, for the reasons mentioned above.

In experiment 4, we investigated whether amnesia following damage that included PRC could be characterized by a heightened susceptibility to perceptual interference. There were three conditions involving High Ambiguity stimuli (Low Interference 1, High Interference, Low Interference 2). The High Interference condition contained consecutive High Ambiguity Object trials, whereas every High Ambiguity Object trial in both Low Interference conditions was interspersed with two trials containing photographs of easily discriminable everyday objects (Figures 2E–2G). We predicted that the nature of the intervening stimuli would affect performance in individuals with PRC damage, with better performance under conditions of low interference.

## Results

### Experiment 1 (Eye Movement Analysis)

**Figure 3:**
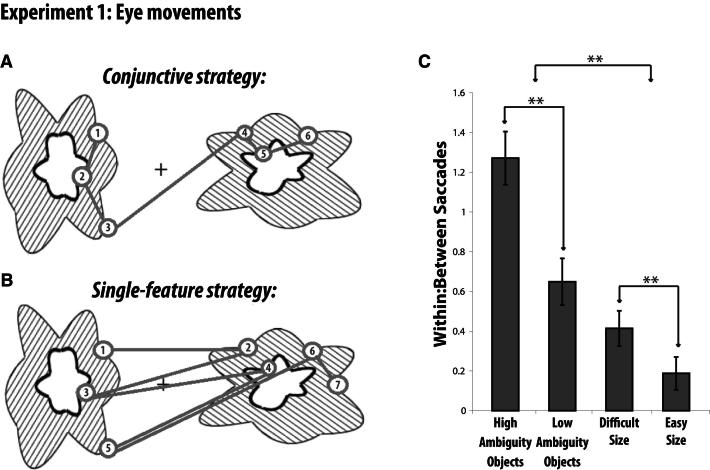
Experiment 1 Example of idealized viewing patterns associated with (A) viewing the stimulus as a whole object (conjunctive strategy) and (B) viewing the stimulus as a series of individual features (single-feature strategy). Each fixation is shown by a numbered circle indicating the order of the fixation; gray lines connecting the fixations indicate saccades. (C) Fixation patterns across the four conditions in experiment 1. The critical ratio of saccades within an item relative to saccades between items indicated that the High Ambiguity Object condition was associated with a greater degree of conjunctive processing. The individual within-item and between-item saccade averages that comprise this ratio are shown in Figure S1. Error bars represent SEM; ^∗∗^p < 0.001.

Analysis of eye movement patterns in healthy participants indicated that the High Ambiguity condition was associated with a greater degree of conjunctive processing than the other conditions. We performed a planned interaction comparison to determine if the High Ambiguity Object condition was associated with more conjunctive processing, relative to our size difficulty control: (High Ambiguity Objects – Low Ambiguity Objects) – (Difficult Size – Easy Size). This revealed that participants made more eye movement transitions within an individual object compared to transitions between the two objects in the High Ambiguity condition relative to the other conditions (t(15) = 4.08; p < 0.001) (Figure 3). Indeed, this ratio of within-item relative to between-item saccades was greater for High compared to Low Ambiguity discriminations (t(15) = 6.58, p < 0.001). We also performed an analysis of the temporal characteristics of these eye movements, which revealed a greater degree of temporal clustering in the High Ambiguity condition (see Supplemental Information, Figure S1C, available online). These results indicate that healthy participants analyzed the ambiguous objects as wholes, rather than by a serial comparison of single features. Experiments 2–4 investigated the neural substrates of this ability.

### Experiment 2 (fMRI)

**Figure 4:**
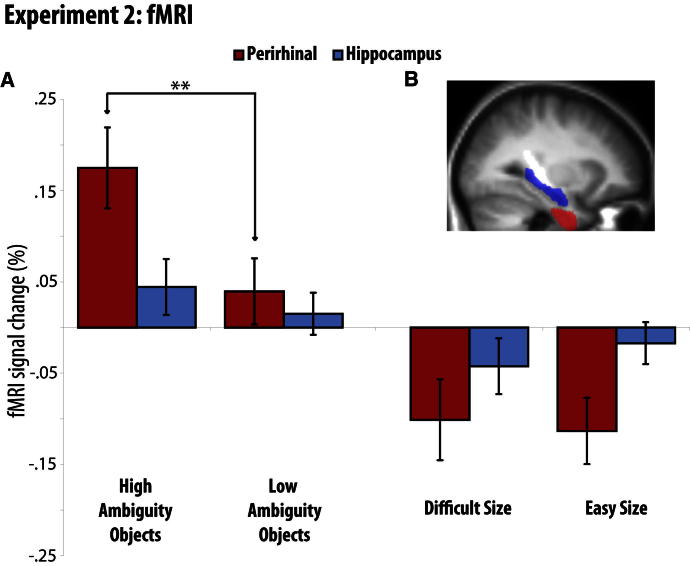
Experiment 2 (A) Percent BOLD change relative to mean over all voxels and scans, mean-corrected over conditions, within the PRC and hippocampal anatomical regions of interest (images were not smoothed). Activity in the PRC was modulated by the degree of feature ambiguity, but not general task difficulty. Activity in the hippocampus was not sensitive to either feature ambiguity or control task difficulty. Error bars represent SEM of the difference between each condition and its relevant control (i.e., High Ambiguity Objects – Low Ambiguity Objects or Difficult Size – Easy Size), ^∗∗^p < 0.001. Accuracy and reaction time data are reported in the Supplemental Information (Table S1). In brief, reduced accuracy was found in High relative to Low Ambiguity conditions, and Difficult relative to Easy control conditions, as expected. Importantly, the planned comparison revealed no greater difference in accuracy between High Ambiguity and Low Ambiguity Objects than between Difficult and Easy Size (t(19) = 0.16), suggesting that fMRI effects of feature ambiguity are not confounded by difficulty. (B) Critical regions of interest superimposed on the mean structural image across participants (PRC in red, hippocampal in blue). See also Figures S2 and S3.

In order to isolate brain regions associated with feature ambiguity resolution, while controlling for general task difficulty, our planned comparison was the same interaction t contrast described above. Estimates of the mean BOLD signal for each of the four conditions were averaged across voxels within our two anatomically defined, bilateral regions of interest: the hippocampus and PRC. The planned comparison revealed feature ambiguity effects within the PRC (t(19) = 3.5, p < 0.001; Figure 4). This region showed reliably greater activity for High relative to Low Ambiguity discriminations (t(19) = 5.2, p < 0.001), but no difference in activity for Difficult relative to Easy Size discriminations (t(19) = 0.5, p = 0.3). By contrast, the comparison of High versus Low Ambiguity Objects was not significant in the hippocampus (t(19) = 1.4, p = 0.1) (and neither was the contrast of Difficult versus Easy Size, t(19) = 1.0, p = 0.2). When activity in the two brain regions was directly compared against one another, we found a significantly greater effect of feature ambiguity in the PRC relative to the hippocampus (t(19) = 4.3, p < 0.001). This finding reflects the first fMRI demonstration of PRC activation during a task in which the critical factor of feature ambiguity (i.e., the presence or absence of repeating features) was precisely controlled.

### Experiments 3 and 4 (Patient Studies)

**Figure 5:**
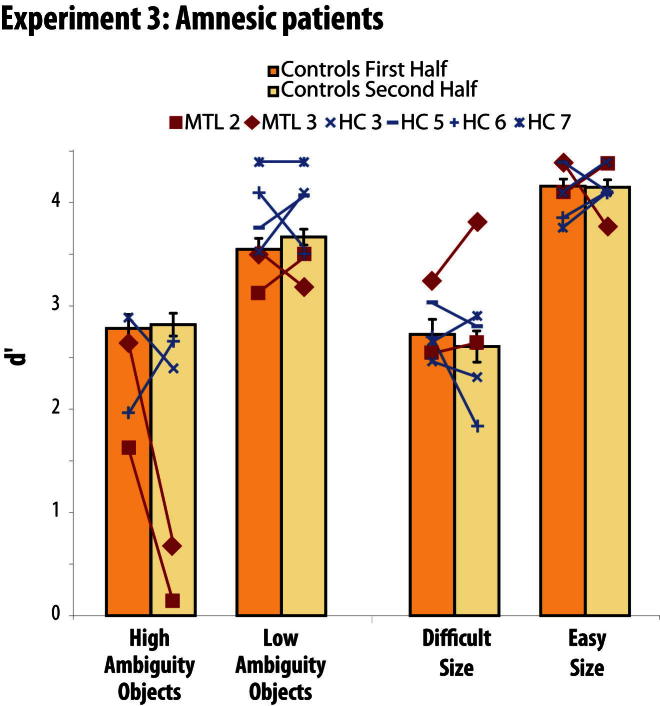
Experiment 3 d′ scores for each individual patient and the mean of their controls, split according to the first and second half of each condition in experiment 3 (patients performed 72 consecutive trials of each condition, with condition order counterbalanced). There was a dramatic decline in performance of patients whose lesion included PRC (MTL cases) as the High Ambiguity condition progressed. This performance decline was limited to the MTL cases on the High Ambiguity Object condition: it was not observed on any other condition or in any other participant group. Moreover, the MTL cases performed normally on the equally challenging Difficult Size condition. Cases with hippocampal lesions (HC cases) performed normally on all conditions. Error bars represent SEM. The separate control groups (age-matched to either the MTL or HC individuals) showed no evidence of differing, in terms of overall performance or relative performance across conditions (all F < 0.7, p > 0.6), and thus are plotted as a single group. A paired t test showed no evidence that control participants found the High Ambiguity Object condition more difficult than the Difficult Size control condition (t(21) = 0.5, p = 0.6), suggesting that the deficit in the MTL cases was not driven by task difficulty. See also Figure S4.

We used Crawford's modified t test to compare each patient to their respective control group (Crawford et al., 2009). Strikingly, we noticed a dramatic drop in performance of both of the MTL cases with PRC damage as the High Ambiguity condition progressed (Figure 5). For the first half (36 trials) of the High Ambiguity Condition, they performed within the normal range (MTL2: t(7) = 1.4, p = 0.1; MTL3: t(7) = −0.1, p = 0.4). By contrast, and inconsistent with traditional accounts of amnesia, for the second half of the condition, their performance fell well below normal performance (MTL2: t(7) = 5.4, p < 0.001; MTL3: t(7) = 4.2, p < 0.01). Critically, this drop in performance was not observed in the individuals with hippocampal lesions (t(7) < 1.0, p > 0.2), nor was it observed on any other condition in either group (t(7) < 1.3, p > 0.1). These findings suggest that the perceptual impairments of the MTL cases with PRC damage arose from the administration of multiple consecutive object discrimination trials, which results in a buildup of interference between shared features. This increased interference can no longer be overcome when conjunctive representations are unavailable, due to PRC damage.

**Figure 6:**
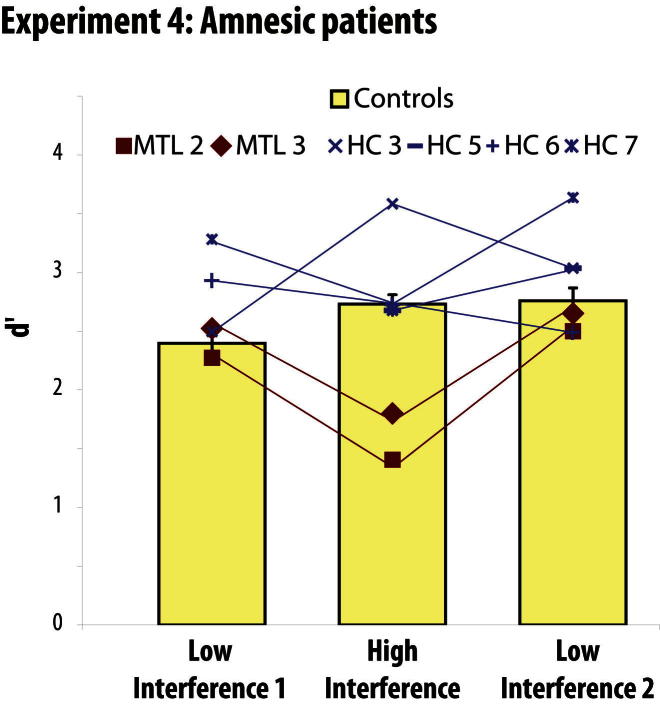
Experiment 4 d′ scores for each individual patient and the mean of their controls in experiment 4. Patients whose lesions included PRC (MTL cases) were impaired on the High Interference condition, but their performance was rescued by reducing the degree of interference. Cases with hippocampal lesions (HC cases) performed normally on all conditions. Error bars represent SEM. The separate control groups showed no evidence of differing, in terms of overall performance or relative performance across conditions (all F < 1, p > 0.4), and thus are plotted as a single group. See also Figure S4.

If this interference hypothesis is correct, we predicted that performance of the MTL cases with PRC damage should improve if we reduced the overlap in features across successive trials. This prediction was confirmed in experiment 4: the two MTL cases with PRC damage were again impaired on the High Interference condition that resembled the High Ambiguity condition of experiment 3 (MTL 2: t(7) = 3.3, p < 0.01; MTL 3: t(7) = 2.4, p < 0.05) (Figure 6), but when we experimentally reduced interference by interspersing dissimilar object trials, we recovered their performance to normal levels (all t(7) < 1.1, p > 0.2). Importantly, in both Low and High Interference conditions, we compared performance on every third trial only (30 High Ambiguity Object comparison trials for each condition). Thus, the important difference across the conditions was the nature of the accumulated perceptual interference across successive trials, not the total number of trials. The intact performance of the MTL cases with PRC damage on the 30 critical High Ambiguity trials in the Low Interference condition is consistent with their performance in experiment 3 (where their deficit emerged after 36 consecutive trials). Only in the High Interference condition, in which the 30 critical High Ambiguity trials were surrounded by twice as many, other interfering (High Ambiguity) trials, did their deficit arise. Furthermore, their intact performance on the Low Interference conditions, particularly the second Low Interference condition, demonstrates that their deficits were specific to the buildup of interfering features, rather than fatigue or generic task-practice effects. The hippocampal cases were not impaired on any condition (all t(7) < 0.4, p > 0.3).

### Analysis of Task Difficulty (Experiments 1–4)

To address the potential concern that differences in task difficulty across conditions could have confounded our results, we analyzed the accuracy and reaction time data of control participants (shown in Figures 5 and 6; Tables S1 and S7; all reported t tests are two-tailed). Importantly, the planned interaction contrast revealed no greater difference in d′ between High Ambiguity and Low Ambiguity Objects than between Difficult and Easy Size (the interaction was not significant in experiment 2, t(19) = 1.1, p = 0.3, and was driven by a bigger drop in performance for Difficult than Easy size conditions in experiments 1 and 3, both t > 2.0, p < 0.06). In experiment 4, the condition on which the MTL patients were impaired (High Interference) was not the condition that controls found to be the most difficult: the High Interference condition was matched in difficulty to Low Interference 2 (t(21) = 0.3, p = 0.8) and significantly easier than the Low Interference 1 (t(21) = 3.1, p < 0.01). These results suggest that our observed eye movement patterns (expt 1), fMRI effects of feature ambiguity (expt 2), and patient deficits (expts 3–4) were not due to global differences in task difficulty.

In terms of reaction times, the increase in RTs for High Ambiguity versus Low Ambiguity Objects was significantly greater than the increase for Difficult versus Easy Size in experiments 1–3 (a trend in expt 1: t(15) = 1.9, p = 0.07; expts 2 and 3: both t > 2.2, p < 0.05). In experiment 4, reaction times for the High Interference condition were significantly longer relative to the second Low Interference condition (t(21) = 3.0, p < 0.01), but were not significantly different from the first Low Interference condition (t(21) = 1.5, p = 0.2). These results suggest that at least for experiment 4, differences in reaction times cannot explain the patients' deficits.

Nonetheless, the finding that in experiments 1–3 the High Ambiguity Object conditions were associated with longer reaction times relative to the Size Control conditions merits further consideration in light of the idea that working memory demands may have differed across conditions. Several studies have reported impairments of short-term memory in amnesia (e.g., Hannula et al., 2006; Nichols et al., 2006; Olson et al., 2006; Warren et al., 2010, 2011, 2012), and neuroimaging studies have observed hippocampal activity in tasks typically considered to assess short-term memory (e.g., Cabeza et al., 2002; Cashdollar et al., 2009; Hannula and Ranganath, 2008; Karlsgodt et al., 2005; Ranganath and D'Esposito, 2001; Stern et al., 2001; Toepper et al., 2010). These studies have largely emphasized hippocampal—not PRC—contributions to working memory, which is not immediately consistent with the intact performance of the individuals with selective hippocampal damage reported here. Nonetheless, it seems likely that the conjunctive representations contained in PRC are essential to maintain information while shifting attention from one complex object to the other. It is important to note, however, that other studies have demonstrated that PRC damage impairs complex object perception on tasks with no working memory component (e.g., perception of single objects), suggesting the deficits observed here are unlikely to be due entirely to working memory (Barense et al., 2011b; Lee and Rudebeck, 2010). That said, both perception and online maintenance of complex objects require the ability to represent conjunctions of object features, and thus, impoverished representations will cause deficits in both processes. As such, we prefer to consider these findings in terms of a representational deficit, rather than a deficit in a given psychological construct (e.g., working memory versus perception).

## Discussion

Here, across four experiments, we present results from a perceptual discrimination task that was shown with eye tracking to emphasize processing conjunctions of object features (experiment 1) and with fMRI to recruit the PRC (experiment 2). Individuals with MTL damage that included the PRC, but not those with damage limited to the hippocampus, were impaired on this task (experiment 3). Critically, when we minimized perceptual interference by reducing the number of repeating features across successive trials, we recovered performance of the MTL cases to normal levels (experiment 4). In contrast to conventional accounts of MTL amnesia, the performance of the MTL cases with PRC damage reported here offers the somewhat paradoxical conclusion that intact memory for irrelevant, lower-level features processed on previous trials can impair *perception* in cases with *memory* disorders. These data are thus not consistent with the view of the MTL as a unitary, dedicated memory system. The data are, however, perfectly consistent with the predictions of the representational-hierarchical theory, which states that the PRC is necessary for representing the conjunctions of features that distinguish perceptually similar objects. These representations become especially critical when the capacity of more posterior regions in the ventral visual stream is exceeded by presentations of multiple, similar features across trials. Indeed, these data provide the first conclusive evidence from humans to complement the related findings from rat lesion studies and computational modeling: namely, that performance of individuals with PRC damage can be rescued by reducing the degree of perceptual interference (Bartko et al., 2010; Burke et al., 2010; Cowell et al., 2006; McTighe et al., 2010; see also Romberg et al., 2012).

The representational-hierarchical theory emphasizes the importance of the organization of representations in a hierarchical continuum throughout the ventral visual processing stream (Cowell et al., 2010b). Under this view, anterior regions such as the PRC contain complex conjunctive representations (e.g., object ABC), whereas more posterior regions contain representations of lower-level features (e.g., features A, B, and C) (Figure 1). At the beginning of the High Ambiguity condition in experiment 3, individuals with PRC damage may have successfully used a single-feature strategy, supported by intact regions posterior to their damage (by definition, the objects in the discrimination of ABC versus ABD contained a single unambiguous feature: C versus D). However, as the condition progressed, more and more perceptually similar features were processed and represented in these posterior regions. Over time (after approximately 36 trials), irrelevant single features from previous trials created interference, and the single-feature strategy became less successful. Whereas individual object features were very similar from trial-to-trial, the objects themselves were trial unique and could be uniquely represented by an intact PRC. The cases with MTL damage including PRC, however, lacked these unique conjunctive PRC representations to disambiguate the single features, and thus, impairments emerged relative to controls and relative to individuals with a damaged hippocampus but an intact PRC. Intermixing perceptually dissimilar objects rather than perceptually similar objects in experiment 4 minimized the degree of interference. When the same number of stimuli were interspersed as in experiment 3—but the stimuli were perceptually dissimilar rather than perceptually similar—the MTL cases were no longer impaired. However, once consecutive trials involving perceptually similar stimuli were introduced, the deficit re-emerged. Thus, we propose that the present findings, and related ones in the animal literature, are best explained in terms of a *representational deficit*, rather than an impairment in a given psychological process, be it memory or perception. Impoverished representations will lead to deficits in all of these processes, and thus, a representational account may provide a more parsimonious explanation for the deficits observed on a wide range of tasks—both mnemonic and perceptual.

Interestingly, although cases with MTL damage including PRC were impaired, cases with selective hippocampal lesions performed normally on the present tasks. This suggests that the effect of interference is dependent on which MTL region is damaged and the specific stimuli that are used. Thus, although vulnerability to object-based perceptual interference may explain visual memory impairments in some cases of MTL amnesia, it is not a general mechanism underlying visual memory impairments in all cases. This is, however, clearly predicted by the representational-hierarchical theory. On this view, PRC contains complex conjunctive representations that specify unique objects, which protects control participants from interfering feature ambiguity. The hippocampus sits even higher in the representational hierarchy, and is necessary for binding object representations (e.g., in PRC) to spatial/temporal representations (Barense et al., 2010a; Bussey and Saksida, 2005; Cowell et al., 2010a; Lee et al., 2005a, 2005b; see also Diana et al., 2007). As such, in situations in which not just features but also objects are repeatedly presented, the representations in PRC would not be enough to protect the participant from interference; the resolution of ambiguity at this level would require conjunctive representations of a higher degree of complexity, such as object representations combined to form spatial “scenes.” We hypothesize that such representations exist in the hippocampus (Lee et al., 2005a; Lee et al., 2005b).

In sum, the present data illustrate how the representational-hierarchical theory offers a promising account of the mechanisms underlying “forgetting” in MTL amnesia, and demonstrate that mnemonic and perceptual impairments following PRC damage can both be explained by an increased vulnerability to object-based perceptual interference. These findings challenge prevailing conceptions of amnesia, suggesting that effects of damage to specific MTL regions are better understood not in terms of damage to a dedicated declarative memory system, but in terms of impoverished representations of the stimuli those regions maintain.

## Experimental Procedures

### Experiment 1: Eye Movement Monitoring

#### Participants

Seventeen undergraduate students (mean age = 21.3 years; SD = 0.5; 11 females) from the University of Toronto participated for either course credit or $10. Due to a computer malfunction, responses from one participant were not recorded on the Easy Size condition and data from this participant was excluded. The age range of the remaining 16 participants (11 female) was 18–23 years (mean age = 21.0 years; SD = 0.4). This experiment received ethical approval from the Ethics Review Office at the University of Toronto.

#### Stimuli and Task

Participants indicated via a button press whether two simultaneously presented trial-unique items were the same or different. The stimuli used for each condition are described below.

#### Feature Ambiguity: Objects

Two abstract objects (similar to the blobs in Barense et al., 2005) were presented on each trial. Each object was placed in one of two nonvisible frames (500 × 500 pixels) that were positioned in the middle of the screen separated by a gap of 8 pixels. The objects subtended a horizontal visual angle ranging from 5.45°–9.07° and a vertical visual angle ranging from 5.5°–9.15°. The object stimuli were always defined by three features: inner shape, outer shape, and fill (Figure 2). Eight different fill features were used and counterbalanced across stimuli. The whole inner and outer shapes were trial unique, but segments of the shapes—as in particular arcs—repeated across trials. For the *High Ambiguity nonmatch* trials, one of the features was changed across the two stimuli while the remaining two features were held constant (e.g., ABC versus ABD; the differing feature was fully counterbalanced). For the *Low Ambiguity nonmatch* trials, none of the three features overlapped across the two objects (e.g., ABC versus DEF). For the *match* trials, the stimuli were identical (ABC versus ABC). For all trials, the objects were rotated by a random number between 15° and 165° to ensure that the exact position of features on the screen was not identical across the two objects.

#### Control Condition: Size

On each trial, two squares were presented. As with the objects, each square was positioned in one of two nonvisible frames separated by 8 pixels, and rotated by a random number between 15° and 165°. The size of each square was trial unique and subtended horizontal and vertical visual angles ranging from 1.45°–13.83°. The position of the squares in the frame was jittered slightly so that the edges of the squares did not line up across horizontal or vertical planes. For *Difficult nonmatch* trials, the length of each side of the square was randomly varied from 67 to 247 pixels. The difference between the lengths of the two squares varied between 9 and 15 pixels (similar to Barense et al., 2010a). By means of several pilot experiments, the difficulty of this condition was designed to closely match that of the High Ambiguity Object condition. For *Easy nonmatch* trials, the length of each side was randomly varied from 40 to 268 pixels, and the difference between the lengths of the two different sides varied between 16 and 40 pixels. Through several pilot experiments, the difficulty of this condition was designed to closely match that of the Low Ambiguity conditions of the Object stimuli. For *match* trials, the two rotated squares were identical in size.

#### Behavioral Procedure

After obtaining informed consent, each of the four conditions (High Ambiguity Objects, Low Ambiguity Objects, Difficult Size, Easy Size) was administered in a fully blocked design, with 72 consecutive trials per condition (36 match trials, 36 nonmatch trials). No feedback was given. Before each condition a short practice (with feedback) of 6 trial-unique stimuli (3 match, 3 nonmatch) was administered. The different conditions were presented in a pseudorandom order, with half the participants receiving the High Ambiguity Object condition prior to the Low Ambiguity Object condition and half the participants receiving the Difficult Size condition prior to the Easy Size condition. The experiment was self-paced, with a maximum of 15 s allowed for each trial. Eye movements were measured using a SR Research Ltd. Eyelink 1000 eye-tracking desktop monocular system and sampled at 1,000 Hz with a spatial resolution of approximately 0.01°.

#### Eye Movement Analysis

The goal of this experiment was to provide evidence into participants' underlying strategy for solving the discriminations. For example, if participants viewed the objects as a conjunction of features, we would expect more saccades *within* an individual object compared to saccades *across* the two objects. By contrast, if participants treated the objects as three separate individual features, we would expect to see more serial comparisons of features across the two objects (Figures 3A and 3B). We predicted that the High Ambiguity condition would place a greater emphasis on the conjunctive strategy than the other conditions, and thus, would be associated with more saccades within single objects relative to saccades between different objects when compared to the other conditions. Because there was no difference between a High Ambiguity match trial and a Low Ambiguity match trial (both involve two identical stimuli), match trials for all conditions were excluded from the analysis. The critical eye movement measures were the number of transitions made by the eyes *across* the two objects (between-item saccades) and the number of transitions made *within* an individual object (within-item saccades) (Figure 3C, see Supplemental Information). We computed the ratio of within-item saccades to total saccades for each trial separately and then averaged the ratios for all 72 trials within each condition for each participant separately. If we let W_i_ be the number of within-item saccades in trial i, and T_i_ be the total number of saccades in trial i, then our ratio is given by

$${\left(\frac{Within}{Total}\right)}_{Av}=\frac{\frac{W_{1}}{T_{1}}+\frac{W_{2}}{T_{2}}+\frac{W_{3}}{T_{3}}+\cdots +\frac{W_{72}}{T_{72}}}{72}$$

We followed the same procedure for between-item saccades. If we let B_i_ be the number of within-item saccades in trial i, and T_i_ be the total number of saccades in trial i, then our ratio is given by

$${\left(\frac{Between}{Total}\right)}_{Av}=\frac{\frac{B_{1}}{T_{1}}+\frac{B_{2}}{T_{2}}+\frac{B_{3}}{T_{3}}+\cdots +\frac{B_{72}}{T_{72}}}{72}$$

Finally, to obtain an estimate of within-item saccades relative to between-item saccades, we divided these two measures to create a ratio for each condition within each participant separately:

$$Within:Between=\frac{{\left(Within/Total\right)}_{Av}}{{\left(Between/Total\right)}_{Av}}$$

We then performed a planned interaction comparison to determine if the High Ambiguity Object condition was associated with more conjunctive processing, relative to our size difficulty control: (High Ambiguity Objects – Low Ambiguity Objects) – (Difficult Size – Easy Size).

To further ensure that any reliable interactions resulting from this predefined comparison were not driven by baseline effects (i.e., interactions driven by the Difficult versus Easy Size comparison as opposed to the High versus Low Ambiguity comparison), we also tested the simple effect of High versus Low Ambiguity, to ensure that it was also reliable. Given our directional hypotheses, all t tests were one-tailed unless stated otherwise.

### Experiment 2: fMRI Study

#### Participants

Twenty-one right-handed healthy participants with normal vision were scanned (14 female, mean age = 22.9 years; SD = 3.2). The data for one participant was excluded because of poor behavioral performance (accuracy more than two standard deviations outside the group mean), possibly due to involvement in a biking accident immediately prior to testing. The age range of the remaining 20 participants (13 female) was 18–29 years (mean age = 22.8 years; SD = 3.2). This experiment received ethical approval from a Cambridgeshire Local Research Ethics Committee.

#### Behavioral Procedure

Participants completed the four conditions from experiment 1 (Figure 2) while undergoing fMRI scanning. The procedure was identical to experiment 1 in nearly every respect, except that we did not monitor eye movements and made some minor modifications so that the paradigm was more suitable for fMRI. In the scanner, the objects subtended a horizontal visual angle ranging from 2.46°–4.10° and a vertical visual angle ranging from 2.51°–4.19°; the squares subtended horizontal and vertical visual angles ranging from 0.66°–6.34°. There were 108 trials for each condition (72 nonmatch and 36 match trials), evenly distributed across four EPI sessions. Each condition was presented in a miniblock of 3 trials of the same condition, and the order of miniblocks (conditions) was chosen in order to maximize the efficiency of fMRI contrasts across conditions (Josephs and Henson, 1999). Within each miniblock, there was always at least one nonmatch trial (i.e., there could have been 1, 2, or 3 nonmatch trials). The assignment of conditions to miniblocks was counterbalanced across participants. Each trial lasted 5.75 s (5.5 s stimulus display time, 0.25 s interstimulus interval). Two short practice sessions with feedback (one outside and one inside the scanner) were administered prior to the start of scanning. Participants were explicitly informed of the ratio of match to non-match trials. In addition to object and size conditions, there were also two conditions consisting of pictures of simple rooms involving a distance judgment between two cones that were designed for a different experimental question.

#### Image Acquisition

Scanning was performed using a Siemens 3T TIM Trio. Four sessions were acquired for every participant. For each data set, an echo planar imaging (EPI) sequence was used to acquire T2^∗^-weighted image volumes with blood oxygen level-dependent (BOLD) contrast. Because temporal lobe regions were the primary area of interest, thinner slices (32 axial-oblique slices of 2 mm thickness) were used in order to reduce susceptibility artifacts (interslice distance 0.5 mm, matrix size 64 × 64, in-plane resolution 3 mm × 3 mm, TR = 2,000 ms, TE = 30 ms, flip angle = 78°). The slices were acquired in a descending order, angled along the axis of the hippocampus to further reduce susceptibility artifacts in anterior medial temporal structures. Each EPI session was 16.4 min in duration, consisting of 5 dummy scans at the start to allow the MR signal to reach equilibrium, and 475 subsequent data scans. A structural scan was acquired for each participant using an MPRAGE sequence (TR = 2,250 ms; TE = 2.99 ms; flip angle = 9°; field of view = 256 mm × 240 mm × 160 mm; matrix size = 256 mm × 240 mm × 160 mm; spatial resolution = 1 mm × 1 mm × 1 mm). We also acquired a field-map for each participant (TR = 400 ms; TE = 5.19 ms/7.65 ms; flip angle = 60°; field of view = 192 mm × 192 mm; matrix size = 64 mm × 64 mm; spatial resolution = 3 mm × 3 mm).

#### Image Preprocessing

The fMRI data were preprocessed and analyzed using Statistical Parametric Mapping software (SPM5, http://www.fil.ion.ucl.ac.uk/spm/software/spm5/). Preprocessing of the data involved (1) realigning all images with respect to the first image of the first session via sinc interpolation and creating a mean image (motion correction); (2) undistorting the EPI data to correct for magnetic field distortions (Cusack and Papadakis, 2002); (3) correcting all images for differences in slice acquisition time using the middle slice in each volume as a reference; (4) normalizing each participant's structural scan to the Montreal Neurological Institute (MNI) T1 ICBM152 average brain template and applying the resulting normalization parameters to the EPI images. For the whole-image analysis, the normalized images were interpolated to 3 × 3 × 3 mm voxels and smoothed with an 8 mm FWHM isotropic Gaussian kernel (final smoothness of approximately 12.6 × 13.0 × 12.2 mm).

#### fMRI Data Analysis

Following preprocessing, statistical analyses were conducted at the individual participant level. For each condition, there were three trial-types: (1) the correct, nonmatch trials of interest, (2) incorrect trials of no interest, and (3) match trials of no interest (match trials were not of interest because they did not contain a level of ambiguity corresponding to either the High or Low condition). Each trial-type was modeled as a separate regressor within a General Linear Model (GLM), thereby allowing the effects of no interest to be covaried from the effect of interest. Within each regressor, each trial was modeled by convolving an on-off boxcar function with a canonical hemodynamic response function. The duration of each boxcar was equal to the stimulus duration (i.e., 5.5 s). To account for residual artifacts after realignment, an additional regressor was added for each volume during which excessive movement occurred (effectively discounting that volume from the effects of interest (Lemieux et al., 2007)). Excessive movement was defined as a translation of more than 0.3 mm in x, y, or z directions, or a rotation greater than π/90 radians (2°) about any of the three axes, relative to the previous volume. Voxelwise parameter estimates for these regressors (which also included a final constant term) were obtained by restricted maximum-likelihood (ReML) estimation, using a temporal high-pass filter (cutoff 128 s) to remove low-frequency drifts, and modeling temporal autocorrelation across scans with an AR(1) process. Contrast images were then calculated by averaging the parameter estimates for each condition across sessions.

Second-level group analyses were conducted on anatomically-defined regions of interest (ROIs) using the MarsBaR toolbox for SPM5 (http://marsbar.sourceforge.net/). Given the relatively small size and the close proximity of the MTL structures of interest, we used unsmoothed images in order to reduce the inclusion of BOLD signal from nearby structures. The data for the GLM for each ROI represented the average across all voxels within that ROI. The perirhinal ROI was the probability map created by combining the anatomical data of 28 participants in Devlin and Price (2007) and Holdstock et al. (2009) (http://joedevlin.psychol.ucl.ac.uk/perirhinal.php). We included areas that had a 50% or more probability of being perirhinal cortex. The hippocampus ROI was defined based on the anatomical automatic labeling (AAL) atlas (Tzourio-Mazoyer et al., 2002). We report results from the bilateral ROIs in the manuscript (Figure 4) and from each unilateral ROI in the Supplemental Information (Figure S2).

The second-level ROI analysis was designed to test the following two predictions: (1) activity averaged across the perirhinal cortex would be modulated by the degree of feature ambiguity, relative to a difficulty control, and (2) the modulation by feature ambiguity would be greater in the perirhinal cortex than in the hippocampus. These predictions were tested by a one-sample t test versus zero for the planned, directional, interaction contrast described in experiment 1.

The planned interaction contrast was also performed on a voxel-by-voxel basis, to investigate brain regions outside the MTL showing any effects of feature ambiguity. For this whole-image analysis, the first-level (individual participant) GLMs were refit to the smoothed, normalized data instead (with the smoothing helping to accommodate residual individual differences in anatomy after normalization, and also helping to ensure parametric assumptions are met for the voxelwise statistics). The resulting parameter estimate images for the four conditions were then entered into a second-level GLM, together with subject effects, on which the same directional interaction t contrast was performed as above. To further ensure that any reliable interactions resulting from this predefined comparison were not driven by baseline effects (i.e., interactions driven by the Difficult versus Easy Size comparison as opposed to the High versus Low Ambiguity comparison), we also tested the simple effect of High versus Low Ambiguity, and concentrated on regions that showed both a reliable interaction and a reliable simple effect of High versus Low Ambiguity. For maxima outside the MTL, a threshold of p < 0.05, two-tailed and FWE-corrected for the whole brain was applied. The results are listed in Table S2. To illustrate the spatial extent of the PRC activation, we have included the statistical map superimposed on the structural images from five representative participants (Figure S3).

Because the PRC is not the only brain region that shows our planned interaction effect, it is important to note that the MTL patients described in experiments 3 and 4 do not have damage in any of these non-MTL regions (Table S2). Thus, the combination of the pattern of damage in the MTL cases and the fMRI data pinpoint the PRC as the critical region for solving the high ambiguity discriminations. Moreover, there is a large literature involving studies in animals with damage neatly circumscribed to PRC indicating that PRC is the critical region for resolving feature ambiguity (Bartko et al., 2010; Buckley et al., 2001; Bussey et al., 2002, 2003; McTighe et al., 2010). We certainly do not wish to suggest, however, that the PRC is the *only* region in the ventral visual stream that is necessary for perceptual processing. Our claim is that the PRC has an important role in perceptual processing, as does every other region in the ventral visual stream. The specific role that each region plays is dependent on the specific level of stimulus complexity that is represented in that region, with regions early in the ventral visual stream necessary for relatively simple representations such as edges and regions later in the ventral visual stream (such as PRC, but other regions as well) necessary for representations of complex objects. Our critical point is that such representations are organized hierarchically and extend into what has classically been considered the MTL memory system.

### Experiments 3 and 4: Patient Studies

#### Participants

For each amnesic patient and each experiment, eight control participants matched in age and level of education (all p > 0.2) were recruited. These experiments received ethical approval from the Ethics Review Office at the University of Toronto, a Cambridgeshire Local Research Ethics Committee, and an Oxfordshire Research Ethics Committee. The performance of each individual patient was compared to his or her respective control group. Details of each case's etiology, demographics, and performance on an extensive neuropsychological battery are provided in Table S3. Some of these individuals have been described in previous reports, and for consistency, the same labels are used here as those used previously (HC3, MTL2, and MTL 3 described in Barense et al., 2007, 2011b; Lee et al., 2005b). Both groups of patients had severe deficits in episodic memory. For example, both patient groups performed similarly poorly on recall of a story and the Warrington Recognition Memory Test for words. Given that there was a substantial mental rotation component in the task used in the current study, all patients and controls were tested separately on a standard mental rotation task (Shepard and Metzler, 1971). None of the patients were impaired on this task relative to controls. The patients' accuracy for two largest angles of rotation (60° and 80°) was 70.0% (SD = 15.2) and controls' accuracy for these angles of rotation was 72.2% (SD = 11.8).

The structural MRI scans of each patient were analyzed in comparison to neurologically healthy control participants. The results of these analyses have largely been reported elsewhere (Barense et al., 2007; Lee et al., 2005b; Lee and Rudebeck, 2010) and are described in detail in the Supplemental Information (Tables S4–S6; Figure S4). In summary, these revealed that the MTL cases had damage to the perirhinal cortex bilaterally. As is common in amnesic patients with large MTL lesions, they had additional damage to the amygdala, entorhinal cortex, hippocampus, parahippocampal cortex, and temporal pole region. Importantly, there were no significant differences between the MTL cases and controls in other regions, in particular the posterior fusiform gyrus or posterior lateral temporal cortex in either hemisphere, suggesting intact posterior regions known to be important for visual processing. The damage in the HC cases was primarily limited to the hippocampus.

It should be noted that some or all patients may have primary or secondary damage or dysfunction in temporal lobe neocortex that cannot be detected by T1-weighted MRI, but which nonetheless may play a role in the pattern of deficits reported here. However, two of the patients (HC3 and MTL3) have undergone functional neuroimaging, which revealed a normal PPA, FFA, and LOC (Lee and Rudebeck, 2010). Thus, it is unlikely that cortical regions more typically associated with visual processing are damaged in these patients. Their profile of performance is consistent with two convergent lines of research that allow more selective localization of the PRC: (1) animal studies that have demonstrated object discrimination deficits and interference effects after selective PRC damage (Bartko et al., 2010; Buckley et al., 2001; Bussey et al., 2002, 2003; McTighe et al., 2010) and (2) the functional neuroimaging data reported here revealing PRC activity in healthy participants during the present discrimination task (see also Barense et al., 2010a, 2011a; Devlin and Price, 2007; Lee et al., 2008; O'Neil et al., 2009).

#### Behavioral Procedure

The testing procedure in experiment 3 was nearly identical to that described in experiment 1 (Figures 2A–2D), except that we did not monitor eye movements. In experiment 4, participants were administered a visual discrimination task similar to that used in experiment 3. There were three conditions involving trial-unique stimuli (Low Interference 1, High Interference, Low Interference 2), with a short (2–5 min) break in between each condition (Figures 2E–2G). The High Interference condition contained 88 High Ambiguity Object trials (44 match, 44 nonmatch). The Low Interference conditions contained 30 High Ambiguity Object trials (15 match, 15 nonmatch) that were interspersed with two trials containing photographs of easily discriminable everyday objects (58 trials; 29 match, 29 nonmatch). Critically, we compared performance on every third trial only. Thus, our comparison trials in each condition were 30 High Ambiguity Object trials with matched stimulus schedules, allowing us to investigate whether the nature of the intervening stimuli affected performance. In the Low Interference condition, the stimuli presented on non-comparison trials overlapped very little with the stimuli on comparison trials. Thus, the level of interference was much lower, and so we predicted that patients with damage to conjunctive representations should be able to perform better on the Low Interference conditions. Based on the findings from experiment 3, we expected the MTL cases to perform well up to approximately 36 trials (when deficits had emerged in the High Ambiguity condition). Critically, in each Low Interference condition, there were only 30 trials involving the comparison High Ambiguity Object stimuli. Thus, even though the number of intervening stimuli was controlled, there was much less build-up of repeated single features in this condition compared to the High Interference condition. As such, we did not expect impairments on this condition (consistent with their intact performance on the first 36 trials of the High Ambiguity condition in experiment 3).

To counter the claim that any deficits in the High Interference condition were due to participant fatigue, the conditions were always administered in the following order: Low Interference 1, High Interference, Low Interference 2. All other parameters were identical to that described in experiment 3. Due to a response box malfunction, the data from the first Low Interference condition for patient HC5 were lost.

#### Data Analysis

We calculated a discriminability measure (d′) using the method developed for same-different judgments (Macmillan and Creelman, 1991). In this analysis, correct responses of “different” to images that were different were designated as hits, and incorrect responses of “different” to images that were in fact the same were designated as false alarms. Scores of 1.0 or 0.0 for hits and false alarms were subjected to a standard correction whereby half a trial was either subtracted or added to the actual score. Data from each individual patient were compared to his or her respective control group using Crawford's Modified t tests (Crawford et al., 2009). Given our directional hypotheses, all t tests were one-tailed unless stated otherwise. In addition to d′ (Figures 5 and 6) we also report reaction times and percent correct for each experiment (Table S7), as well as percent correct split according to High Ambiguity Object nonmatch trial type (fill, inner shape, outer shape; Table S8).

## Supplemental Information

Document S1. Four Figures, Eight Tables, and Supplemental Experimental Procedures
