# Supplementary material for: Intact Memory for Irrelevant Information Impairs Perception in Amnesia
Source: Neuron. 2012 Jul 12;75(1):157–67. doi: 10.1016/j.neuron.2012.05.014 (PMC3657172; doi:10.1016/j.neuron.2012.05.014)
Supplement: Document S1. Four Figures, Eight Tables, and Supplemental Experimental Procedures [file mmc1.pdf]

Article

Intact Memory for Irrelevant Information

Impairs Perception in Amnesia

Morgan D. Barense, Iris I.A. Groen, Andy C.H. Lee, Lok-Kin Yeung, Sinead M. Brady, Mariella Gregori, Narinder Kapur, Timothy J. Bussey, Lisa M. Saksida, and Richard N.A. Henson

Inventory of Supplemental Information

Supplemental Data: Figures

Figure S1. Fixation patterns in Experiment 1, shown in terms of the average number of within-item saccades and the average number of between-item saccades.

Figure S2. Analysis of BOLD signal change within the PRC and hippocampal regions of interest in Experiment 2, split according to hemisphere.

Figure S3. Regions of PRC activity in the voxel-wise analysis in Experiment 2.

Figure S4. Percent intracranial volume for the participants in Experiments 3 and 4.

Supplemental Data: Tables

Table S1. Average discriminability, percent correct, and reaction times in Experiments 1 and 2.

Table S2. Significant voxels outside the MTL for the planned comparison in Experiment 2.

Table S3. Results from the neuropsychological battery (Experiments 3 and 4).

Table S4. Structural MRI scan ratings for patient MTL2 (Experiments 3 and 4).

Table S5. Individual patient's z-scores in the volumetric analyses (Experiments 3 and 4).

Table S6. Repeatability analyses for the volumetric analyses (Experiments 3 and 4).

Table S7. Average percent correct and reaction times in Experiments 3 and 4, split according to match and non-match trials.

Table S8. Average percent correct in Experiments 3 and 4, split according to which feature differed (fill pattern, inner shape, outer shape).

Supplemental Results and Experimental Procedures

Experiment 1 (Eye movement monitoring):

Description of eye movement monitoring procedures

Within- and between- item saccades

Temporal clustering of eye movements

Experiment 2 (fMRI study):

Analysis of unilateral ROIs

Experiments 3-4 (Patient studies):

Description of methods used in the volumetric assessment of patient lesions

Analysis of volumetric results

Description of behavioral procedures and test order

Supplemental References

Supplemental Information

Supplemental Data: Figures

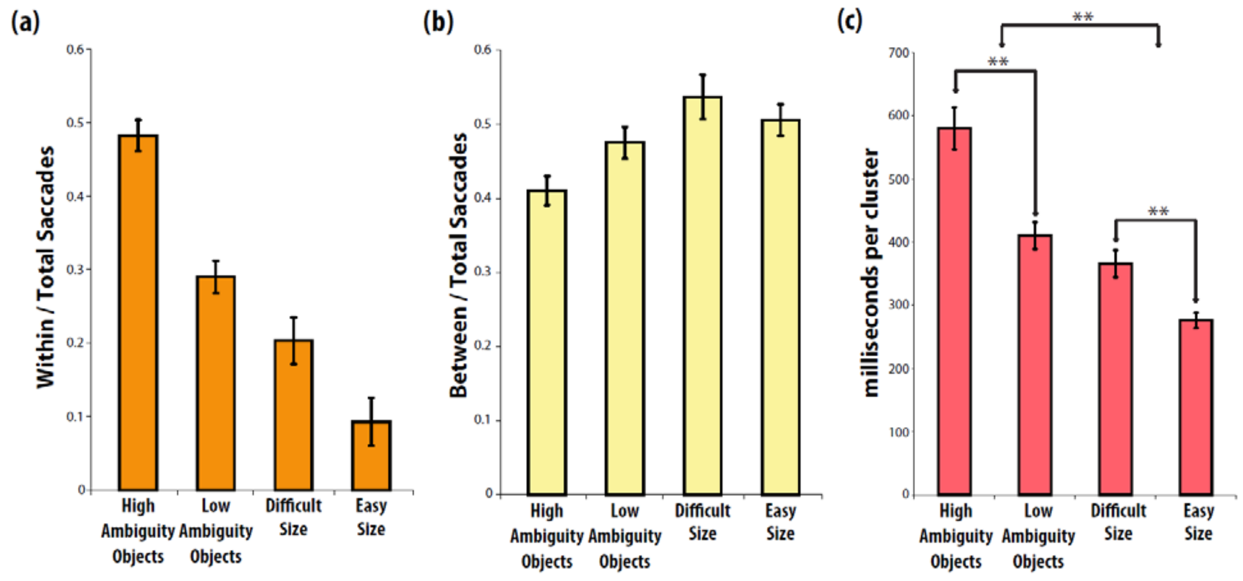

Figure S1 (related to Figure 3). Fixation patterns across the four conditions in Experiment 1. The critical ratio of saccades within an item relative to saccades across items indicated that the High Ambiguity Object condition was associated with a greater degree of conjunctive processing (shown in **Fig. 3** in main text). This within:between saccade ratio was obtained by dividing (a) the average number of within-item saccades by (b) the average number of between-item saccades for a given condition. Note that these figures do not sum to 1 because some fixations landed outside of either stimulus (e.g., the initial fixation on the fixation cross) and thus were not included in the analysis because they were neither within-item saccades nor between-item saccades. **(c)** Average time spent on each cluster of fixations across conditions. Error bars represent SEM, ** $p < 0.01$.

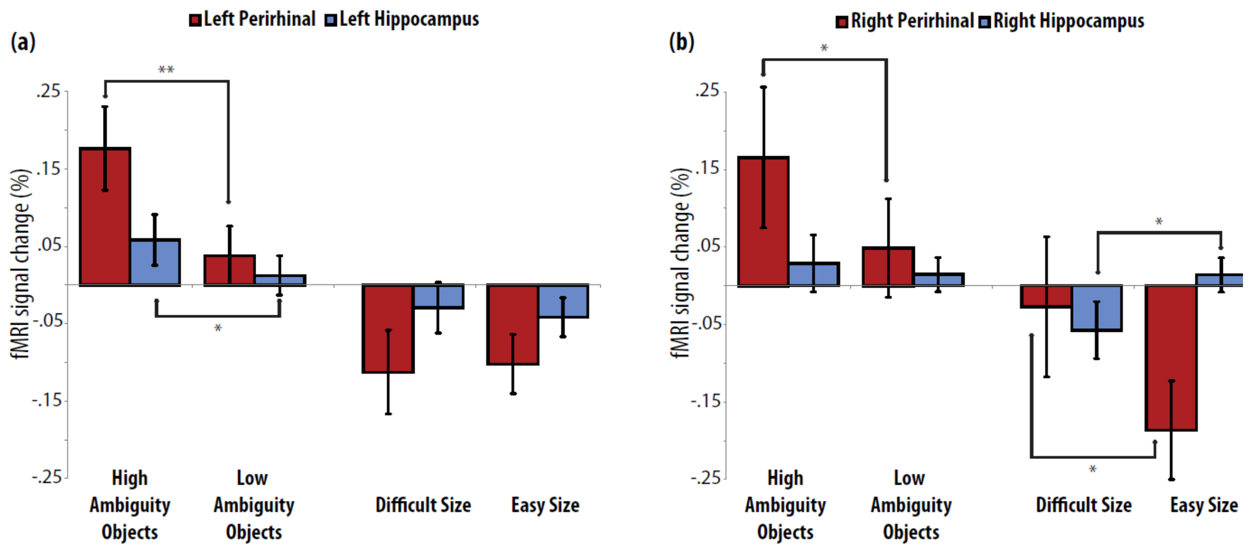

Figure S2 (related to Figure 4). Percent BOLD change relative to mean over all voxels and scans, mean-corrected over conditions within the (a) left and (b) right PRC and hippocampal regions of interest in Experiment 2. Error bars represent SEM of the difference between each condition and its relevant control (i.e., High Ambiguity Objects – Low Ambiguity Objects or Difficult Size – Easy Size), ** $p < 0.001$, * $p < 0.05$.

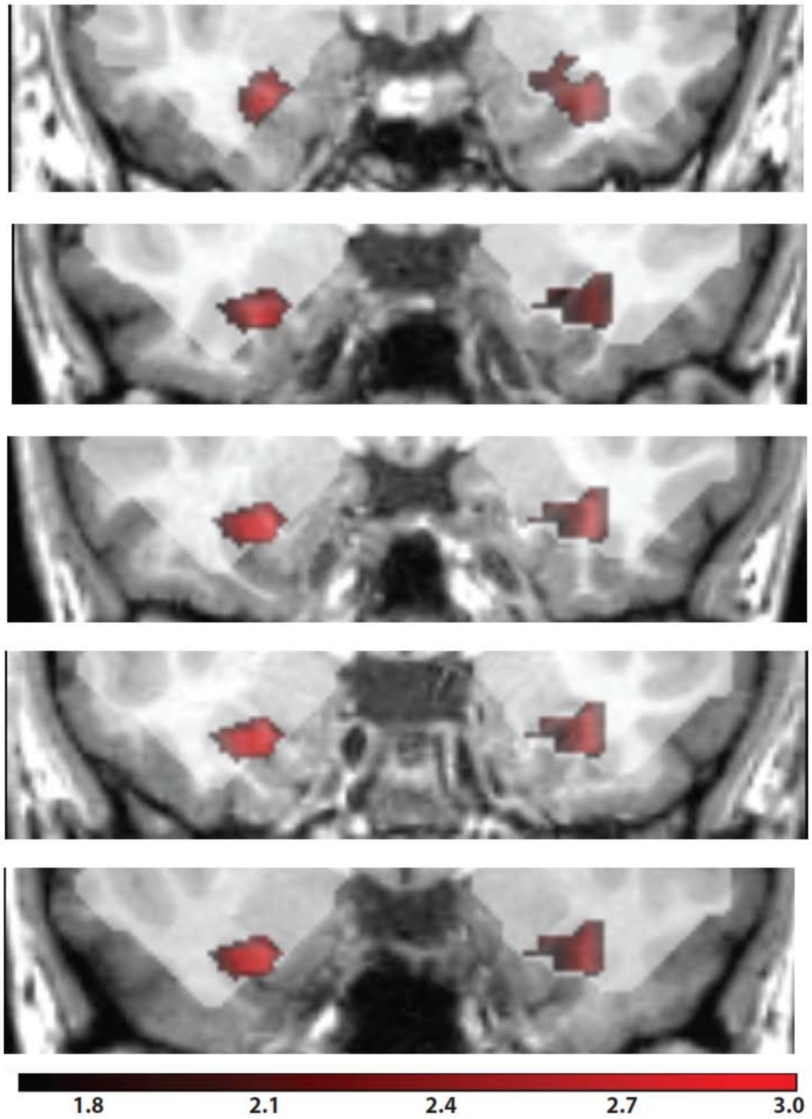

Figure S3 (related to Figure 4). Regions of PRC activity in the voxel-wise analysis for our planned interaction contrast [(High Ambiguity Objects – Low Ambiguity Objects) – (Difficult Size – Easy Size)]. Statistical maps of the group one-tailed T-statistic (df = 19; colour bar inset) are superimposed on the normalized structural images of five representative participants in the present study. To show the spatial extent of the activations, these maps were thresholded at $p < .05$ (uncorrected). To show regions of BOLD signal dropout, the mask image across all participants at the second level is also superimposed (in pale white).

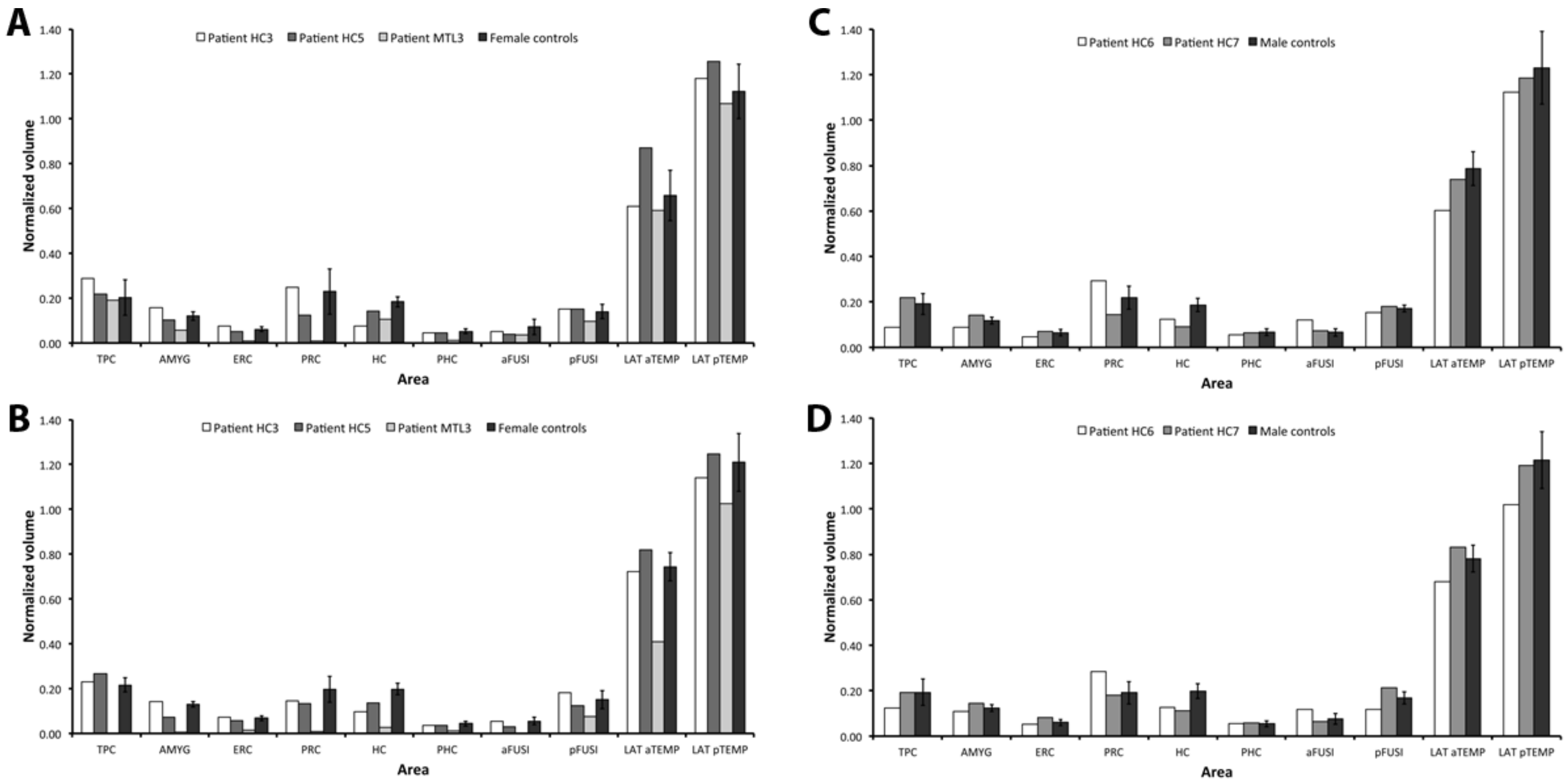

Figure S4 (related to Figures 5 and 6). Normalized volumes (% intracranial volume) for each measured brain region in the (A) left and (B) right hemispheres (±Std Dev) for the female patients and matched female controls and for the (C) left and (D) right hemispheres for the male patients and matched male controls. Key: TPC = temporopolar cortex; AMYG = amygdala; ERC = entorhinal cortex; PRC = perirhinal cortex; HC = hippocampus; PHC = parahippocampal cortex; aFUSI = anterior fusiform gyrus; pFUSI = posterior fusiform gyrus; aLAT TEMP = anterior lateral temporal cortex; pLAT TEMP = posterior lateral temporal cortex.

Supplemental Data: Tables

Table S1. Average discriminability (d'), percent correct, and reaction times for participants in the eye movement (Experiment 1) and fMRI (Experiment 2) studies. Standard deviations shown in parentheses. HA = High Ambiguity; LA = Low Ambiguity.

	**d'**	**Percent correct**		**RT (ms)**	
		**Non-match**	**Match**	**Non-match**	**Match**
**Experiment 1:**					
HA Objects	3.28 (0.83)	90.80 (6.78)	83.51 (12.23)	4595 (1027)	6880 (1833)
LA Objects	4.34 (0.53)	98.96 (1.72)	90.45 (8.60)	1938 (462)	5780 (1845)
Difficult Size	2.81 (0.84)	82.64 (11.41)	81.08 (14.67)	2564 (1024)	3109 (1524)
Easy Size	4.47 (0.65)	97.15 (3.82)	95.65 (4.86)	1231 (245)	2012 (1849)
**Experiment 2:**					
HA Objects	2.27 (0.18)	76.78 (7.80)	77.00 (13.13)	2924 (363)	4007 (297)
LA Objects	3.42 (0.33)	98.13 (3.03)	80.26 (14.77)	1756 (256)	3849 (354)
Difficult Size	2.18 (0.41)	69.70 (16.27)	87.15 (9.18)	2125 (510)	2017 (371)
Easy Size	3.43 (0.33)	97.92 (2.76)	86.82 (11.00)	1314 (267)	1907 (348)

Table S2. Significant voxels outside the MTL for the planned interaction contrast of (High Ambiguity Objects – Low Ambiguity Objects) – (Difficult Size – Easy Size).

Brain Region	***BA***	***p (FWE)***	***Z-stat***	***x***	*y*	*z*
**Left hemisphere**						
Inferior parietal gyrus	40	0.004	5.07	−30	−45	+42
Inferior temporal gyrus	20	<0.001	5.74	−45	−48	−15
Inferior occipital gyrus	37	<0.001	6.11	−48	−60	−12
Middle temporal gyrus	37	0.004	5.06	−48	−63	0
Superior parietal gyrus	7	0.019	4.73	−21	−66	+57
Inferior parietal gyrus	7	0.001	5.37	−27	−66	+45
Middle occipital gyrus	19	0.002	5.26	−27	−72	+30
Precuneus	7	0.001	5.33	−12	−75	+48
Middle occipital gyrus	19	0.011	4.85	−33	−81	+15
**Right hemisphere**						
Thalamus		0.008	4.93	+9	−9	+6
Thalamus		0.001	5.34	+21	−27	+6
Inferior temporal gyrus	20	0.005	5.04	+54	−45	−9
Inferior temporal gyrus	37	0.003	5.12	+51	−54	−9
Angular gyrus	7	0.009	4.89	+30	−60	+42
Superior parietal gyrus	7	0.003	5.14	+27	−63	+51
Precuneus	7	0.004	5.07	+12	−63	+51

There were no suprathreshold clusters for the reverse contrast: (Low Ambiguity Objects – High Ambiguity Objects) – (Easy Size – Difficult Size).

Table S3. Neuropsychological battery. Maximum scores are provided in brackets where applicable. Individual cells for each patient represent raw data scores.

	MTL2	*MTL3*	*HC3*	*HC5*	*HC6*	*HC7*	*Controls (SD)*
Etiology	Viral encephalitis	Viral encephalitis	CO induced hypoxia	Limbic encephalitis	Anoxia during surgery	CO induced hypoxia	
Age	76	63	50	68	56	40	Expt 3: 57.3 (12.0) Expt 4: 58.5 (10.6)
Sex	M	F	F	F	M	M	
Years education	12	10	10	16	13	15	Expt 3: 13.4 (2.9) Expt 4: 13.9 (2.5)
**Recall:**							
WMS III immediate story recall (/75)	29	13	22	34	10		37.1 (9.4)
WMS III delayed story recall (/50)	0	4	4	1	1		20.1 (8.0)
RCF delayed recall (/36)	0	4.5	3	4.5	0		18.4 (5.8)
BMIPB immediate story recall (/60)				18 (<2%ile)	15 (5-10%ile)	5 (<2 %ile)	
BMIPB delayed story recall (/60)				4 (<2%ile)	3 (<2 %ile)	0 (<2 %ile)	
BMIPB complex figure immediate recall (/80)				30 (37.5%ile)	16 (17.5%ile)	38 (37.5%ile)	
BMIPB complex figure delayed recall (/80)				27 (34%ile)	0 (<2 %ile)	13 (16.25%ile)	
**Recognition:**							
WMS III story recognition (/30)	19	23	19	21	17		24.5 (3.1)
WRMT words (/50)	31 (<5%ile)	31 (<5%ile)	33 (<5%ile)	42 (50%ile)	32 (<5%ile)	47 (75%ile)	
WRMT faces (/50)	32 (<5%ile)	30 (<5%ile)	44 (50%ile)	33 (<5%ile)	24 (<5%ile)	42 (25-50%ile)	
**Visuoperceptual:**							
Benton Facial Recognition (/54)	45	42	47	46			Normal: 41-54
VOSP (all sub-tests)	Pass	Pass	Pass	Pass			
RCF copy (/36)	36	30.5	35	32	35		34.0 (1.8)
BMIPB figure copy (/80)				77 (96%ile)	75 (10-25%ile)	80 (75-90%ile)	
**Semantic:**							
Naming (/64)	55	46	64	64			62.3 (1.7)
Graded Naming (/30)	22 (75-50%ile)		21 (75-50%ile)	21 (75-50%ile)	25 (90%ile)	25 (90%ile)	
Word-Picture Matching (/64)	59	54	64	64			63.8 (0.4)
PPT pictures (/52)	49	46	52	51			51.2 (1.4)

**Executive:**							
WCST (categories, /6)	6	6	6		6		5.8 (0.5)
Digit Span – forwards	8	6	6	7	7	6	7.2 (0.9)
Digit Span – backwards	7	4	6	7	5	6	5.3 (1.3)
RCPM (/36)	33 (>95%ile)	22 (50%ile)	34 (>95%ile)	28 (75-90%ile)			

Neuropsychological tests: WMS III = Wechsler memory scale, 3rd edition (Wechsler, 1997); RCF = Rey Complex Figure (Osterrieth, 1944); BMIPB = Brain Injury Rehabilitation Trust Memory and Information Processing Battery (Oddy et al., 2007); WRMT = Warrington Recognition Memory Test (Warrington, 1984); Benton Facial Recognition Test (Benton et al., 1994); VOSP = Visual Object and Space Perception Battery (Warrington and James, 1991); Naming (Adlam et al., 2010); Graded Naming (McKenna and Warrington, 1983); Word-Picture Matching (Adlam et al., 2010); PPT = Pyramids and Palm Trees test (Howard and Patterson, 1992); WCST = Wisconsin Card Sorting Test (Nelson, 1976); RCP = Raven's Colored Progressive Matrices (Raven, 1962). Where percentiles given, norms are based on the test manual. Controls for WMS from Haaland et al. (2003); controls for RCF, Naming, Word-Picture Matching, Digit Span (forwards and backwards) from Adlam et al. (2010), controls for PPT from Hodges et al. (1995); controls for WCST from Graham et al. (2004).

Table S4. Structural MRI scan ratings (with standard deviations) for patient MTL2 and a group of age-matched controls for various brain regions (ordered from anterior to posterior location in the brain), averaged across both hemispheres. Table adapted from Barense et al. (2005).

	Temporopolar cortex	*Amygdala*	*PHG*	*MBCS*	*Perirhinal cortex (LBCS)*	*MBOS*	*Anterior hippocampus*	*Lateral temporal cortex*	*Posterior hippocampus*
MTL2	2*	3*	2.5*	2.75*	2.5*	2*	3*	1	2.75*
Control mean (n=12)	0.313 (0.284)	0.375 (0.483)	0.188 (0.188)	0.521 (0.291)	0.271 (0.310)	0.333 (0.289)	0.458 (0.382)	0.458 (0.411)	0.271 (0.361)

0 indicates no visible damage, 3 (4 for anterior hippocampus) indicates complete absence of area. PHG: parahippocampal gyrus (corresponding to entorhinal cortex); MBCS: medial bank of collateral sulcus (corresponding to the transition between entorhinal and perirhinal cortex); LBCS: lateral bank of collateral sulcus (corresponding to perirhinal cortex); MBOS: medial bank of occipitotemporal sulcus (corresponding to the transition between perirhinal and isocortex. *Significant difference compared with control mean.

Table S5. Individual patient's z-scores for each measured brain region in the left and right hemispheres. Bold text indicates *reduced* volume between individual and matched controls using Crawford's modified t-test. * significant at $p < 0.05$ (two-tailed), + a trend at $p = 0.06$ (two-tailed)

	**Temporopolar cortex**	**Amygdala**	**Entorhinal cortex**	**Perirhinal cortex**	**Hippocampus**	**Parahippocampal cortex**	**Anterior fusiform gyrus**	**Posterior fusiform gyrus**	**Anterior lateral temporal cortex**	**Posterior lateral temporal cortex**
LEFT										
HC3	1.06	1.86	1.44	0.18	**-4.78***	-0.74	-0.57	0.39	-0.43	0.49
HC5	0.21	-0.90	-1.04	-1.04	-1.76	-0.55	-0.93	0.30	1.88	1.12
HC6	**-2.23+**	-1.94	-1.33	1.45	**-2.17+**	-0.73	3.18*	-1.07	**-2.49***	-0.68
HC7	0.59	1.57	0.24	-1.45	**-3.34***	-0.12	0.37	0.74	-0.63	-0.28
MTL3	-0.17	**-3.23***	**-4.72***	**-2.19+**	**-3.46***	**-3.59***	-1.08	-1.36	-0.58	-0.45
RIGHT										
HC3	0.43	0.94	0.31	-0.90	**-3.92***	-0.73	-0.09	0.78	-0.33	-0.53
HC5	1.60	**-4.49***	-1.00	-1.08	**-2.39***	-0.79	-1.51	-0.67	1.17	0.28
HC6	-1.19	-0.98	-0.58	1.91	**-2.19+**	0.11	1.66	-1.92	-1.73	-1.55
HC7	-0.01	1.36	1.51	-0.24	**-2.68***	0.42	-0.43	1.67	0.85	-0.19
MTL3	**-7.01***	**-9.94***	**-4.63***	**-3.21***	**-6.66***	**-2.84***	**-3.31***	-1.87	**-5.27***	-1.41

Table S6. Repeatability analyses across all measured brain regions in male participants using intraclass correlation coefficients. A significant correlation coefficient indicates reliable repeatability. L. = left hemisphere; R. = right hemisphere.

Region	n	coefficient	significance
L. temporopolar cortex	5	0.998	<0.0001
L. amygdala	5	0.986	<0.0001
L. entorhinal cortex	5	0.950	0.003
L. perirhinal cortex	5	0.999	<0.0001
L. hippocampus	5	0.992	<0.0001
L. parahippocampal cortex	5	0.910	0.001
L. anterior fusiform gyrus	5	0.996	<0.0001
L. posterior fusiform gyrus	5	0.938	0.004
L. anterior lateral temporal cortex	5	0.961	0.001
L. posterior lateral temporal cortex	5	0.965	0.001
R. temporopolar cortex	5	0.998	<0.0001
R. amygdala	5	0.940	0.001
R. entorhinal cortex	5	0.940	0.002
R. perirhinal cortex	5	0.985	<0.0001
R. hippocampus	5	0.993	<0.0001
R. parahippocampal cortex	5	0.972	<0.0001
R. anterior fusiform gyrus	5	0.987	<0.0001
R. posterior fusiform gyrus	5	0.962	0.001
R. anterior lateral temporal cortex	5	0.975	0.001
R. posterior lateral temporal cortex	5	0.956	0.001

Table S7. Average percent correct and reaction times for participants in Experiments 3 and 4, split according to match and non-match trials. Standard deviations shown in parentheses. HA = High Ambiguity; LA = Low Ambiguity.

	**Percent correct**		**RT (ms)**	
	**Non-match**	**Match**	**Non-match**	**Match**
**<u>Experiment 3:</u>**				
**HA Objects**				
Controls	85.86 (8.44)	79.42 (11.55)	5027 (1354)	6492 (1722)
HC cases	81.94 (5.89)	77.78 (0.00)	6114 (611)	7924 (1117)
MTL 2	58.33	58.33	6904	6810
MTL 3	75.00	61.11	8636	8526
**LA Objects**				
Controls	95.58 (6.04)	89.02 (7.20)	2530 (882)	5297 (2078)
HC cases	99.31 (1.39)	93.06 (4.81)	2608 (428)	7013 (1212)
MTL 2	91.67	88.89	3690	6478
MTL 3	100.00	77.78	4829	8263
**Difficult Size**				
Controls	75.13 (12.73)	85.61 (12.08)	2567 (689)	2597 (732)
HC cases	72.22 (5.07)	88.19 (6.56)	3726 (1067)	3604 (906)
MTL 2	66.67	91.67	3765	3418
MTL 3	88.89	94.44	5121	3988
**Easy Size**				
Controls	99.24 (1.75)	96.34 (5.9)	1296 (276)	1478 (463)
HC cases	99.31 (1.39)	95.14 (2.66)	1510 (441)	1902 (580)
MTL 2	97.22	100.00	1647	1535
MTL 3	100.00	94.44	2762	3197

	Percent correct		RT (ms)	
	Non-match	Match	Non-match	Match
**<u>Experiment 4:</u>**				
**Low Interference 1**				
Controls	74.48 (12.76)	80.00 (14.40)	5406 (999)	6746 (1867)
HC cases	88.89 (3.85)	82.22 (7.70)	5567 (231)	9168 (963)
MTL 2	86.67	66.67	5462	6758
MTL 3	93.33	60.00	6644	9204
**High Interference**				
Controls	85.15 (8.47)	79.70 (12.08)	4830 (1111)	6641 (1551)
HC cases	88.33 (8.39)	83.33 (3.85)	4933 (978)	7553 (1474)
MTL 2	66.67	60.00	6502	6846
MTL 3	73.33	66.67	6443	8166
**Low Interference 2**				
Controls	71.39 (15.94)	91.21 (10.96)	4728 (1303)	5282 (1690)
HC cases	88.33 (10.00)	85.00 (6.38)	5372 (719)	7090 (1153)
MTL 2	93.33	60.00	7530	9561
MTL 3	66.67	93.33	5757	5871

Table S8. Average percent correct for High Ambiguity non-match trials in Experiments 3 and 4 split according to which feature differed (fill pattern, inner shape, outer shape). Standard deviations shown in parentheses. HA = High Ambiguity.

	**Fill**	**Inner**	**Outer**
**<u>Experiment 3:</u>**			
**HA Objects**			
Controls	91.67 (8.91)	90.91 (8.10)	74.24 (18.17)
HC cases	75.00 (11.79)	87.50 (5.89)	83.33 (0.00)
MTL 2	33.33	75.00	66.67
MTL 3	58.33	75.00	91.67
**<u>Experiment 4:</u>**			
**Low Interference 1**			
Controls	84.09 (0.25)	80.00 (0.16)	62.88 (0.23)
HC cases	91.67 (0.14)	93.33 (0.12)	83.33 (0.00)
MTL 2	50.00	100.00	100.00
MTL 3	100.00	80.00	100.00
**High Interference**			
Controls	87.12 (0.17)	87.27 (0.15)	79.55 (0.24)
HC cases	87.50 (0.08)	85.00 (0.10)	93.75 (0.13)
MTL 2	33.33	100.00	75.00
MTL 3	66.67	60.00	100.00
**Low Interference 2**			
Controls	83.64 (0.23)	78.18 (0.28)	59.05 (0.19)
HC cases	90.00 (0.12)	95.00 (0.10)	80.00 (0.16)
MTL 2	100.00	100.00	80.00
MTL 3	80.00	40.00	80.00

Supplemental Results and Experimental Procedures

Experiment 1: Eye movement monitoring

Testing and eye movement monitoring procedures

Stimuli were displayed on a flat-screen 19” CRT monitor at a resolution of 1024x768 and participants indicated their response via the keyboard (‘n’ for non-match and ‘m’ for ‘match’). Failure to respond within the allotted timeframe was counted as an error and resulted in the onset of the next trial. Participants were instructed to respond as quickly but as accurately as possible, and there were very few trials on which participants timed-out. Between trials there was a 0.5 second inter-stimulus-interval during which a white screen was presented. A nine-point calibration was performed at the start of every condition followed by a nine-point calibration accuracy test. Calibration was repeated if the average gaze error was greater than 1° and if the error at any single point was more than 1.5°. A chin rest positioned 55 cm from the screen was used to limit head movements.

Based on our predictions, analysis of eye movements was performed with respect to areas of interest created for each individual stimulus. For the object stimuli, these areas of interest were two adjacent 320 x 320 pixel squares located in the center of the screen and separated horizontally by 508 pixels. To ensure that any fixations on the edge of the object were included in our analyses, the area of interest always contained the entire object plus at least 15 pixels of blank space in all directions. Because the square stimuli differed in size across trials, areas of interest for the squares were more specifically tailored to the size of individual stimuli in order to minimize the amount of blank space captured within the areas of interest. Thus, these areas of interest were variable in size but were always rectangular and were never closer than 15 pixels from the edge of any square.

Results: Eye movements within versus between objects

The ratio of within:between item saccades reported in **Figure 3c** was obtained by dividing the average number of within-item saccades by the average number of between-item saccades for a given condition. These within-item and between-item saccade averages are shown in **Figure S1**. These data provide objective evidence that healthy participants process the High Ambiguity Objects as a conjunction of features, rather than a series of single features. We have shown with fMRI that this process recruits the PRC (**Experiment 2**) and that this ability is impaired by brain damage that includes the PRC (**Experiment 3**).

Results: Temporal clustering of eye movements

In addition to comparing saccades within objects relative to saccades between objects, we also analyzed the temporal characteristics of saccade clusters made within stimuli on non-match trials. We defined a cluster of fixations as successive fixations made to the same stimulus without any fixations outside that stimulus. For example, **Figure 3a** represents two clusters (fixations 1-3 would form one cluster and fixations 4-6 would form a second cluster), whereas Figure 3b represents six clusters (the individual fixations 1, 2, 3, 4, and 5 each comprise a separate cluster, and fixations 6-7 comprise a cluster). For each cluster in each condition across all participants, we calculated the time from the first fixation of a cluster to the last fixation of that cluster (i.e., the amount spent on each cluster). We then averaged time spent on clusters for each condition and each participant separately and performed our planned interaction comparison [(High Ambiguity Objects – Low Ambiguity Objects) – (Difficult Size – Easy Size)]. This revealed more temporal clustering in the High Ambiguity Object condition, relative to the size difficulty control ($t(15) = 2.9$, $p < 0.05$). The simple

effect of High versus Low Ambiguity was also significant ($t(15) = 9.3$, $p < 0.001$), indicating that the interaction was not driven by baseline effects. Data are displayed in **Figure S1c.**

Experiment 2: fMRI study

Results: Unilateral ROI analysis

To complement the analyses that collapsed across hemisphere in the main paper, we also performed our analyses on unilateral ROIs for the hippocampus and PRC. Estimates of the mean BOLD signal for each of the four conditions were averaged across voxels within both the left and right ROIs. Both the left and right PRC showed reliably greater activity for High relative to Low Ambiguity discriminations (both $t(19) > 2.1$, $p < 0.05$). The control comparison of Difficult versus Easy Size discriminations was not significant in the left PRC ($t(19) = 0.4$, $p = 0.4$), but it was significant in the right PRC ($t(19) = 2.7$, $p < 0.01$). As such, the planned interaction was significant in the left PRC ($t(19) = 3.5$, $p < 0.001$), but not in the right PRC ($t(19) = 0.5$, $p = 0.3$). In the left hippocampus, the comparison of High versus Low Ambiguity Objects was significant ($t(19) = 2.8$, $p < 0.01$), but the planned interaction contrast ($t(19) = 1.0$, $p = 0.2$) and the Difficult versus Easy Size contrast were not ($t(19) = 0.4$, $p = 0.3$). In the right hippocampus the comparison of High versus Low Ambiguity Objects was not significant ($t(19) = 0.5$, $p = 0.3$), whereas the contrast of Difficult versus Easy size was significant ($t(19) = 2.7$, $p < 0.01$). Nonetheless, the numerical pattern of results was very similar across left and right ROIs (**Fig. S2**). There were no significant hemispheric differences for the contrast of High versus Low Ambiguity Objects in either the PRC or hippocampus (both $t(19) < 1.5$, $p > 0.14$, two-tailed). Both the right hippocampus and PRC showed greater activity (compared to the left hippocampus and PRC, respectively) for the baseline comparison of Difficult versus Easy Size, although in different directions (right HC driven by more activity for Easy Size ($t(19) = 3.5$, p

< 0.01, two-tailed; right PRC driven by more activity for Difficult Size (t(19) = -2.6, p < 0.05, two-tailed). There was no significant interaction between hemisphere and our planned comparison in the hippocampus (t(19) = -1.6, p = 0.13, two-tailed). In the PRC there was a trend for a greater effect in the left than in the right hemisphere (t(19) = 1.8, p = 0.09, two-tailed), but this was clearly driven by baseline differences, not by ambiguity differences. As such, there is no clear evidence for important functional differences between hemispheres in either the hippocampus or PRC.

Experiments 3-4: Patient studies

Volumetric assessment of patient lesions

The structural MRI scans of each patient were analyzed in comparison to either matched female or male neurologically healthy control participants. Due to claustrophobia, it was not possible to obtain a research-quality structural MRI scan for Patient MTL2 that was suitable for volumetric analyses. Nonetheless, qualitative visual ratings of a previous clinical MRI scan of patient MTL2 revealed significant damage to the hippocampus, amygdala, anterior temporal cortex, medial and lateral bank of the collateral sulcus and the medial bank of the occipitotemporal sulcus, but not the lateral temporal cortex (described in Barense et al., 2007; Lee et al., 2005) (**Table S4)**. This method has been validated successfully against volumetric measures (Galton et al., 2001).

The volumetric data for Patients HC3 and MTL3 and 11 matched female control participants (mean age 55.27 years, SD = 10.80) are taken from a previous study (Lee and Rudebeck, 2010). The structural scan of Patient HC5 (256 x 122 x 256 in size, voxel dimensions 0.86 x 1.80 x 0.86 mm) was acquired on a 1.5T GE Signa scanner at the MRI Department, Addenbrooke's Hospital, Cambridge, UK and compared to the same female control data (no significant difference in age between Patient HC5 and controls, t(10) = 0.68, p = 0.51). ROIs were manually traced on coronal

slices in each hemisphere using MRIcron software (Rorden and Brett, 2000) and previously published methods (Lee and Rudebeck, 2010). The hippocampus and amygdala were defined with the Mayo Clinic method (Watson et al., 1997), whereas the temporopolar cortex, entorhinal cortex, and perirhinal cortex were identified using the Insausti protocol (Insausti et al., 1998). The parahippocampal cortex was measured from the slice following the posterior boundary of the perirhinal cortex, and the fusiform gyrus was measured from the slice coinciding with the anterior boundary of the perirhinal cortex. The posterior boundaries of both the parahippocampal cortex and fusiform gyrus coincided with the posterior boundary of the hippocampus. A measure for lateral temporal cortex was obtained by measuring the grey matter of the entire temporal cortex from the tip of the temporopolar cortex to the posterior end of the hippocampus and subtracting the volumes for temporopolar cortex, entorhinal cortex, perirhinal cortex, parahippocampal cortex, and the fusiform gyrus. Because the anterior portion of the temporal lobe toward the temporal pole is not known to be associated with visual processing (i.e., as part of the ventral visual processing stream), the fusiform gyrus and lateral temporal volumes were subdivided in two by measuring separately the slices anterior and posterior to the midpoint. All measured volumes were corrected for (scaled by) intracranial volume, which was determined by drawing around the brain tissue in all coronal slices including gray and white matter, ventricular space, and excluding the brainstem below the level of the pons.

It is important to acknowledge that damage to temporal lobe regions more traditionally associated with perception, such as areas TE/TEO as identified in nonhuman primates, could present a confound in the interpretation of our findings. Although there is no clear consensus on exactly what regions in the human brain correspond to areas TE/TEO in the macaque brain (Seltzer and Pandya, 1978; Von Bonin and Bailey, 1947), it is likely that these regions are captured by the

lateral temporal lobe rating (**Table S4**), and the posterior lateral temporal cortex and posterior fusiform gyrus volumes (**Table S5**) (Hadjikhani et al., 1998; Sewards, 2011). Critically, the MTL cases did not show significant atrophy in these regions. Patient MTL3 did possess significantly lower scores on the anterior lateral temporal cortex and anterior fusiform gyrus measures in the right hemisphere (**Table S5**), due largely to an absent right temporal pole. It seems unlikely, however, that areas TE/TEO are located within this temporal pole region (Hadjikhani et al., 1998; Sewards, 2011). Critically, in our fMRI experiment we did not observe any significant activity associated with feature ambiguity resolution in the temporal pole area (even at an uncorrected threshold of $p < 0.05$), further suggesting that Patient MTL3's damage to this area is not responsible for her deficits.

The structural scans for Patients HC6 and HC7, and 10 matched male controls (mean age 47.90 years, SD = 12.02) were acquired on a 1.5T GE Signa scanner at the MRI Department, Addenbrooke's Hospital, Cambridge, UK or a 3T Siemens TRIO at the MRC CBU, Cambridge, UK. All scans were 256 x 122 x 256 in size, with voxel dimensions 0.86 x 1.80 x 0.86 mm. ROIs were manually traced according to the same methods described above. Repeatability in the male participants was assessed by re-measuring all ROIs in 5 of the cases at least 6 weeks after the first measurement (1 patient and 4 controls) and calculating intra-class correlation coefficients. Good repeatability was found in all areas (all $r > 0.9$; see **Table S6**).

Volumetric results

Individual patient's z-scores for each measured brain region are reported in **Table S5**. As reported elsewhere (Lee and Rudebeck, 2010), there were no significant differences between HC3 and female controls in any region (all $-0.9 < t(10) < 1.8$, $p > 0.1$), other than the left ($t(10) = -4.57$, p =

0.001) and right hippocampus ($t(10) = -3.76$, $p = 0.004$) (**Fig. S4a-b**). There were no significant differences between HC5 and female controls in any region (all $-1.8 < t(10) < 1.9$, $p > 0.1$), other than the right hippocampus ($t(10) = -2.39$, $p = 0.04$) and right amygdala ($t(10) = -4.49$, $p = 0.002$) (**Fig. S4a-b**). HC6 showed a trend towards significantly reduced hippocampus bilaterally (both $t(9) = 2.1$, $p = 0.06$) (**Fig. S4c-d**). In addition, HC6 had significantly greater volume in the left anterior fusiform gyrus ($t(9) = 3.04$, $p = 0.01$), and significantly less volume in the left anterior lateral temporal cortex ($t(9) = -2.37$, $p = 0.04$), as well as a trend for less volume in the left temporopolar cortex ($t(9) = -2.13$, $p = 0.06$). There were no significant differences in any other region (all $-1.9 < t(10) < 1.9$, $p \geq 0.1$). There were no significant differences between HC7 and male controls in any region (all $-1.4 < t(10) < 1.7$, $p > 0.1$), other than the left ($t(9) = -3.19$, $p = 0.01$) and right hippocampus ($t(9) = -2.55$, $p = 0.03$) (**Fig. S4c-d**).

As reported previously (Lee and Rudebeck, 2010), MTL3 has damage to the perirhinal cortex bilaterally (right perirhinal cortex: $t(10) = -3.07$, $p = 0.01$; left: $t(10) = -2.10$, $p = 0.06$). As is common in amnesic patients with large MTL lesions, patient MTL3 has additional damage to the amygdala, entorhinal cortex, hippocampus, and parahippocampal cortex bilaterally (all $t(10) < -2.7$, $p < 0.03$), as well as the temporopolar cortex, anterior fusiform gyrus, and anterior lateral temporal cortex in the right hemisphere (all $t(10) < -3.1$, $p < 0.02$) (**Fig. S4a-b**). Importantly, there were no significant differences between MTL3 and female controls in other regions, in particular the posterior fusiform gyrus or posterior lateral temporal cortex in either hemisphere (all $t(10) > -1.8$, $p > 0.1$), suggesting intact posterior visual regions and lateral temporal areas.

Behavioral procedure and test order

All tests were conducted on a 15'' laptop at a resolution of 1024x768. Participants made their responses by pressing a custom-made two-button box that was connected to the laptop through a USB-port. The buttons were labeled such that patients could always see which button corresponded to which answer. The experiments were programmed using E-Prime software (Psychology Software Tools Inc., Pittsburgh, PA). The different conditions of Experiment 3 were presented in a pseudorandom order, with half the patients receiving the High Ambiguity Object condition prior to the Low Ambiguity Object condition and half the patients receiving the Difficult Size condition prior to the Easy Size condition (the MTL cases received opposite orders). Controls completed the conditions in the same order as the patient to whom they were matched. Patients MTL2, MTL3, and HC3 completed Experiment 3 six months prior to Experiment 4. HC6 completed Experiment 4 one month prior to Experiment 3. Due to their limited availability, HC5 and HC7 completed Experiments 3 and 4 in the same testing session, with Experiment 4 administered prior to Experiment 3. Because the High Ambiguity Object condition of Experiment 3 is essentially identical to the High Interference condition of Experiment 4, these patients did not complete the High Ambiguity Object condition. Given their normal performance on the High Interference condition, it seems reasonable to assume that they would have also performed normally on the High Ambiguity Object condition.

Supplemental References

Adlam, A.L., Patterson, K., Bozeat, S., and Hodges, J.R. (2010). The Cambridge Semantic Memory Test Battery: detection of semantic deficits in semantic dementia and Alzheimer's disease. Neurocase *16*, 193-207.

Barense, M.D., Bussey, T.J., Lee, A.C., Rogers, T.T., Davies, R.R., Saksida, L.M., Murray, E.A., and Graham, K.S. (2005). Functional specialization in the human medial temporal lobe. J Neurosci *25*, 10239-10246.

Barense, M.D., Gaffan, D., and Graham, K.S. (2007). The human medial temporal lobe processes online representations of complex objects. Neuropsychologia *45*, 2963-2974.

Benton, A.L., Sivan, A.B., Hamsher, K., Varney, N.R., and Spreen, O. (1994). Contributions to Neuropsychological Assessment (New York: Oxford University Press).

Galton, C.J., Gomez-Anson, B., Antoun, N., Scheltens, P., Patterson, K., Graves, M., Sahakian, B.J., and Hodges, J.R. (2001). Temporal lobe rating scale: application to Alzheimer's disease and frontotemporal dementia. J Neurol Neurosurg Psychiatry *70*, 165-173.

Graham, N.L., Emery, T., and Hodges, J.R. (2004). Distinctive cognitive profiles in Alzheimer's disease and subcortical vascular dementia. J Neurol Neurosurg Psychiatry *75*, 61-71.

Haaland, K.Y., Price, L., and Larue, A. (2003). What does the WMS-III tell us about memory changes with normal aging? J Int Neuropsychol Soc *9*, 89-96.

Hadjikhani, N., Liu, A.K., Dale, A.M., Cavanagh, P., and Tootell, R.B. (1998). Retinotopy and color sensitivity in human visual cortical area V8. Nat Neurosci *1*, 235-241.

Hodges, J.R., and Patterson, K. (1995). Is semantic memory consistently impaired early in the course of Alzheimer's disease? Neuroanatomical and diagnostic implications. Neuropsychologia *33*, 441-459.

Howard, D., and Patterson, K. (1992). Pyramids and palm trees: A test of semantic access from pictures and words (Bury St Edmunds, England: Thames Valley Test).

Insausti, R., Juottonen, K., Soininen, H., Insausti, A.M., Partanen, K., Vainio, P., Laakso, M.P., and Pitkanen, A. (1998). MR volumetric analysis of the human entorhinal, perirhinal, and temporopolar cortices. AJNR Am J Neuroradiol *19*, 659-671.

Lee, A.C., Bussey, T.J., Murray, E.A., Saksida, L.M., Epstein, R.A., Kapur, N., Hodges, J.R., and Graham, K.S. (2005). Perceptual deficits in amnesia: challenging the medial temporal lobe 'mnemonic' view. Neuropsychologia *43*, 1-11.

Lee, A.C., and Rudebeck, S.R. (2010). Human medial temporal lobe damage can disrupt the perception of single objects. J Neurosci *30*, 6588-6594.

McKenna, P., and Warrington, E.K. (1983). The graded naming test (Windsor, Berkshire: NFER-Nelson).

Nelson, H.E. (1976). A modified card sorting test sensitive to frontal lobe defects. Cortex *12*, 313-324.

Oddy, M.O., Coghlan, A.K., and Crawford, J.R. (2007). BIRT Memory and Information Processing Battery (Horsham, UK: Brain Injury Research Trust).

Osterrieth, P.A. (1944). Le test de copie d'une figure complexe. Archives de Psychologie *30*, 205-220.

Raven, J.C. (1962). Coloured progressive matrices sets A, AB, B (London: H.K. Lewis).

Rorden, C., and Brett, M. (2000). Stereotaxic display of brain lesions. Behav Neurol *12*, 191-200.

Seltzer, B., and Pandya, D.N. (1978). Afferent cortical connections and architectonics of the superior temporal sulcus and surrounding cortex in the rhesus monkey. Brain Res *149*, 1-24.

Sewards, T.V. (2011). Adolf Hopf's 1954 myeloarchitectonic parcellation of the human temporal lobe: a review and assessment. Brain Res Bull *86*, 298-313.

Von Bonin, G., and Bailey, P. (1947). The neocortex of Macaca mulatta (Urbana, IL: University of Illinois).

Warrington, E.K. (1984). The recognition memory test (Windsor: NFER-Nelson).

Warrington, E.K., and James, M. (1991). Visual Object and Space Perception Battery (VOSP) (Oxford: Harcourt Assessment).

Watson, C., Jack, C.R., Jr., and Cendes, F. (1997). Volumetric magnetic resonance imaging. Clinical applications and contributions to the understanding of temporal lobe epilepsy. Arch Neurol *54*, 1521-1531.

Wechsler, D. (1997). Wechsler Memory Scale. Third edition manual. (San Antonio: The Psychological Corporation).
